# A mechanistic study of the dopant-induced breakdown in halide perovskites using solid state energy storage devices†

**DOI:** 10.1039/d2ee01754g

**Published:** 2022-09-06

**Authors:** Angus G. M. Mathieson, Wesley M. Dose, Hans-Georg Steinrück, Christopher J. Takacs, Sascha Feldmann, Raj Pandya, Alice J. Merryweather, David Mackanic, Akshay Rao, Felix Deschler, Michael De Volder

**Affiliations:** Institute for Manufacturing, Department of Engineering, University of Cambridge 17 Charles Babbage Rd Cambridge CB3 0FS UK mfld2@cam.ac.uk; Cavendish Laboratory, Department of Physics, University of Cambridge 17 JJ Thomson Ave Cambridge CB3 0HE UK; Cambridge Graphene Centre, Department of Engineering, University of Cambridge 9 JJ Thomson Ave Cambridge CB3 0HE UK; Yusuf Hamied Department of Chemistry, University of Cambridge Lensfield Road Cambridge CB2 1EW UK; Department Chemie, Universität Paderborn 33098 Paderborn Germany; SSRL Materials Science Division, SLAC National Accelerator Laboratory Menlo Park California 94025 USA; Rowland Institute, Harvard University Cambridge Massachusetts 02142 USA; Physikalisch-Chemisches Institut, Universität Heidelberg, Im Neuenheimer Feld 229 69120 Heidelberg deschler@uni-heidelberg.de

## Abstract

Doping halide perovskites (HPs) with extrinsic species, such as alkali metal ions, plays a critical, albeit often elusive role in optimising optoelectronic devices. Here, we use solid state lithium ion battery inspired devices with a polyethylene oxide-based polymer electrolyte to dope HPs controllably with lithium ions. We perform a suite of *operando* material analysis techniques while dynamically varying Li doping concentrations. We determine and quantify three doping regimes; a safe regime, with doping concentrations of <10^20^ cm^−3^ (2% Li : Pb mol%) in which the HP may be modified without detrimental effect to its structure; a minor decomposition regime, in which the HP is partially transformed but remains the dominant species; and a major decomposition regime in which the perovskite is superseded by new phases. We provide a mechanistic description of the processes mediating between each stage and find evidence for metallic Pb^(0)^, LiBr and LiPbBr_2_ as final decomposition products. Combining results from synchrotron X-ray diffraction measurements with *in situ* photoluminescence and optical reflection microscopy studies, we distinguish the influences of free charge carriers and intercalated lithium independently. We find that the charge density is equally as important as the geometric considerations of the dopant species and thereby provide a quantitative framework upon which the future design of doped-perovskite energy devices should be based.

## Abstract 2

Elucidating and quantifying the effects of doping on halide perovskites using lithium ion batteries.

## Graphical abstract

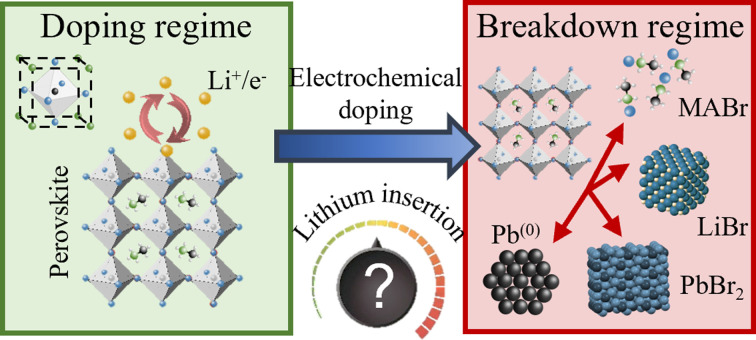

**Broader context**

Hybrid perovskites are arguably one of the most promising material systems for the next generation of energy devices, with reports of record-breaking energy conversion efficiencies occurring on an almost yearly basis. However, unlike traditional semiconductors such as silicon and GaAs, the impact of external dopants in these materials remains poorly understood. There currently exist relatively few reliable methods with which to insert extrinsic species into the perovskite lattice and an experimental model describing the impact on the perovskite structure has remained somewhat elusive. This work represents a breakthrough in understanding what happens to hybrid perovskites when Li-ions are added into the lattice at dopant concentrations that span three orders of magnitude. This is achieved by a battery-inspired device architecture, which allows one to vary the dopant concentration in a perovskite semiconductor, while simultaneously facilitating *operando* synchrotron XRD and optical analysis. The findings of this work have general implications in both the energy conversion and energy storage communities by quantifying three doping thresholds in hybrid perovskites, within which the doping can be achieved with either reversible or irreversible changes to the perovskite structure.

## 1. Introduction

Halide perovskites (HPs) have generated considerable interest as the base material for a myriad of optoelectronic devices.^1^ The high absorption coefficients, long carrier lifetimes and high (and balanced) hole and electron mobilities make them extremely promising in the development of the next generation of optoelectronic and photovoltaic devices.^2,3^ At the time of writing, the state-of-the-art perovskite solar cell can generate a power conversion efficiency of 25.7%.^4^ This is notable primarily due to its contradiction to the previously held assumption that inherent imperfections in a solution-processed semiconductor, such as a HP, would result in poor performance (relative to the highly crystalline Si & GaAs technologies) due to excessive non-radiative recombination and poor charge transport.^5^ This resilience of the HP class of materials to defects has resulted in the emergence of manifold optoelectronic devices, first and foremost the solar cell,^3,6–8^ additionally, lasers,^9,10^ light emitting diodes (LEDs)^11–15^ and photodetectors.^16–19^ The versatility of this class of materials is demonstrated in their recent developments in fields outside of the photovoltaic and opto-electronics communities, namely in gas sensing,^20^ data storage,^21^ radiation detectors,^22,23^ computing,^24^ and thermo-electrics.^25^ However, in addition to the main focus of HP research, a rapidly growing area of research exists in their application as electrodes in energy storage devices, such as metal-ion batteries^26–30^ and light rechargeable photobatteries.^31^

**Fig. 1:**
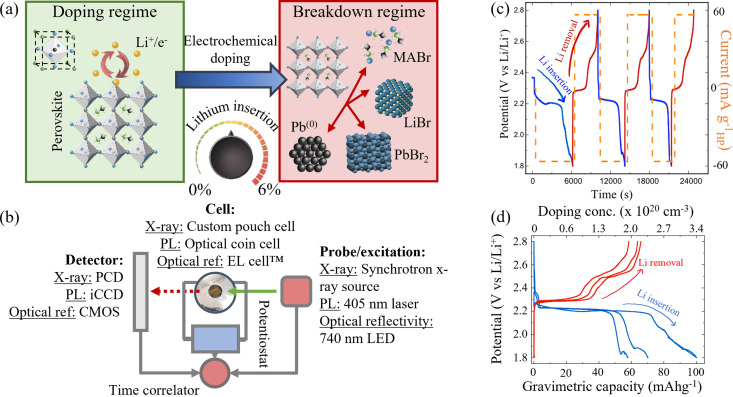
(a) Schematic representation of two doping regimes. Left: Safe doping regime, where Li is reversibly inserted into the perovskite structure. (MAPbBr_3_ unit cell shown inset). Right: Breakdown regime with phase conversion products shown inset. (b) Schematic reduction of the *operando* characterisation techniques used to probe the properties of the perovskite while dynamically inserting and removing Li ions *via* electrochemical cycling. PCD – Dectris PILATUS 300 kW photon counting detector. iCCD – intensified charge coupled device. CMOS – complementary metal oxide semiconductor active pixel sensor. (c) Galvanostatic cycling process used to insert (blue traces) and remove (red traces) lithium from MAPbBr_3_. The potential *vs.* Li/Li^+^ varies as lithium is inserted and removed (left axis). Right axis (orange trace) indicates the applied current at each stage. (d) Charge–discharge curves of the first three insertion and removal cycles at 60 mA g^−1^. Gravimetric capacity corresponds to the amount of Li^+^ inserted (blue) or removed (red) in each cycle.

The perovskite class of materials now encompasses a great variety of structures and chemical compositions. Methylammonium (MA) lead iodide (CH_3_NH_3_PbI_3_) is perhaps the most commonly recognised composition and engenders the standard three-dimensional (3D) perovskite ABX_3_ structure (unit cell as shown inset in Fig. 1(a)). In this instance, A and B represent primary and secondary cations respectively with the A cation generally being a monovalent organic cation, such as MA or formadinium, and B a divalent inorganic cation – oftentimes lead, but also tin or germanium. X represents the halide anion species, typically iodide, bromide, chloride (I^−^, Br^−^, Cl^−^) or mixtures thereof. Compositional engineering allows for the design and synthesis of a plethora of materials, which despite sharing a common structure, can vary significantly in physical properties and applications. The ability to change the absorption coefficient, optical bandgap (over an extensive range of wavelengths extending beyond the visible),^3,32–34^ and improve stability against various environmental species such as water and oxygen^35–37^ through a simple alteration of the elemental composition is one of the characteristics of the perovskite portfolio making them so effective in a great variety of disciplines.

Furthermore, by inserting a primary A cation that is too large to fit directly within the bulk perovskite crystal structure, it is possible to form a quasi two-dimensional (2D) layered species of the form A_large_BX_4_, where A_large_ denotes the larger cationic species such as butylammonium (BA).^38,39^ In doing so, upon reaching a truly 2D interlayer limit, electronic quantum confinement effects result in further modifications to the electronic structure and therefore optical bandgap of the material to tailor the characteristics of the material.^34,40–42^ Furthermore, due to the increased hydrophobicity of the longer organic cation, it is possible to increase the native stability of the material against atmospheric conditions.^43,44^

For optoelectronic and photovoltaic applications, the doping of external species into HPs has received considerable attention,^45–47^ but has to some extent remained experimentally elusive. It has not been possible to dope perovskites using the established techniques of the inorganic semiconductor industry – such as ion implantation in crystalline silicon^48^ – and has therefore required the development of new techniques. Some examples include ground-state molecular charge transfer – which has been shown as a viable technique to dope perovskite nanocrystals and quantum dots owing to their high surface area to volume ratio.^49^ However, although effective in these low dimensional class of perovskites, the technique is not suitable for thin film devices. Substitutional techniques, such as replacing the A site cation with EA^+^ (ethyl ammonium), Sc^+^, Ag^+^, *etc.* have been demonstrated as a viable alternative in the bulk materials.^50^ However, most substitutional techniques rely on in synthesis processes, which depending on the annealing and crystallisation conditions lead to a variable quality and homogeneity in the dopants.

In addition to the substitution of A or B ions in the HP, adding species that sit interstitially within the structure have recently been shown to engender interesting material effects. For example, the insertion of ions with an active spin, such as manganese, has been shown to create magnetically active HP semiconductors.^51,52^ The insertion of Li has also been proposed to improve the performance of LED devices by tailoring the energy levels at the interface between the charge insertion layers and the emitting perovskite layer, thus improving the electron insertion efficiency and reducing the turn-on voltage.^14,53^ The findings of this work aim to provide a quantitative framework within which these device performance enhancements may be harnessed.

Furthermore, the insertion of alkali metal ions into perovskites has been demonstrated to stabilise luminescence and reduce non-radiative losses when inserted in small quantities (0–10% by molar ratio Li : Pb) by quenching non-radiative trap states inherent to the material.^46,54^ We also demonstrated in earlier work that electrochemically doping MAPbBr_3_ in concentrations up to the order of 10^19^ cm^−3^, can result in an n-type doping of the material.^55^ This is due to the fact that each Li ion inserted using this technique must necessarily correspond to the insertion of an electron from the external battery circuit, which we have shown to be inserted into the conduction band of the HP.^47,55^ It is clear therefore, that the careful control of dopants in a HP thin film is of the utmost importance to improving the performance metrics of HP solar cell and LED devices – by mitigating the effects of inherent atmospheric dopants, but also tailoring HPs to a specific doping state for integration into full solar cell or LED device stacks. In this current work, we use a similar technique and increase the lithium doping concentration within the HP to levels far exceeding the previous study and in doing so, elucidate the resilience of HPs to external species and investigate the phase transformation processes that occur once the limit has been exceeded.

Reports on lithium insertion in perovskites also include the work of Cao *et al.*^56^ and Dawson *et al.*^57^ Cao *et al.* demonstrate how the incorporation of extrinsic alkali cations (of which Li is included) occupy interstitial sites, resulting in the suppression of halide migration within the perovskite layer. Dawson *et al.* demonstrate how Li ions can be incorporated interstitially into the perovskite layer, when used as an electrode in a lithium ion battery (LIB) device and use density functional theory to determine which conversion reactions are energetically favourable upon excessive electrochemical discharge. In the current work, we manage to provide experimental verification of the theoretically proposed mechanisms of Dawson *et al.*^57^ The work of Dawson *et al.* also calculates the possible structural distortions due to Li insertion of all three of the commonly studied HP halide chemistries, namely I, Br, and Cl based perovskites. A difference in the electrochemical behaviour of I^−^ compared to Br^−^-based HPs can also be found in a previous study.^31^ The ionic nature of the bonding within the HP crystal structure renders it inherently sensitive to the insertion of external ions (compared to layered metal oxide materials employed in LIB electrodes for example), and only so many dopants may be inserted interstitially before the structure breaks apart.^57^ Previous research of HPs for LIBs has shown that lithiating HPs leads to a range of conversion and alloying reactions.^27,57^

In this work, Li is incorporated into the host material lattice in quantities ranging from 0.2–20 mol% and the effects of doing so, including the phase conversion reactions that take place at excessive concentrations are elucidated. To achieve this, we use an electrochemical cell resembling the architecture of an LIB, equipped with a window making the cells suitable for both *operando* X-ray diffraction (XRD) and optical measurements. The advantage of electrochemical doping is that the number of Li ions inserted into the HP can be calculated and controlled – simply by controlling the current flow through the cell and the time, since it is known that 1 Coulomb of charge measured through the external circuit represents 6.24 × 10^18^ Li ions added to the HP. However, side reactions can introduce errors and the electrolyte needed to transport the Li ions from the Li source (here a Li-metal foil) typically rely on polar solvents that tend to dissolve the HP over time. In this work however, we address this issue by using a polymer-based solid state electrolyte and demonstrate a stable HP/electrolyte interface – allowing the influence of the intercalating Li to be studied directly and without interference from dissolution.

We define and quantify three doping regimes: a safe regime, a minor decomposition regime, and a major decomposition regime. In addition to identifying each doping regime, we elucidate the mechanisms that mediate between them and determine the phase conversion processes responsible for the loss of perovskite structure at high dopant concentrations. This provides a framework and upper ceiling of attemptable doping concentrations for future perovskite device modification schemes. Finally, we utilise both the 3D and 2D perovskite compositions and mixtures thereof for generality and in order to isolate and probe a variety of crystallographic structures and ensure the universality of the results. The Ruddlesden Popper quasi 2D/3D mixed phase, of the form (BA)_2_(MA)_*n*−1_Pb_*n*_X_3*n*+1_ (BA – butylammonium, MA – methylammonium, X – halide (I^−^ and Br^−^)) with *n* = 4 (mixed phase) and *n* = ∞ (pure 3D) phases are studied and changes to the diffraction spectra at dynamically varying Li doping concentrations are shown. The loss in PL intensity is used to isolate the effects of the doping on the perovskite electronic energy structure – such as the introduction of trap states and majority charge carrier quenching – from the structural and phase conversion effects deduced from the XRD study. Finally, by looking at the loss in optical reflectivity from a perovskite crystallite, the lithium doping and phase conversion dynamics are characterised, demonstrating an outward-in procession of diminishing perovskite phase fraction.

## 2. Results and discussion

Galvanostatic (constant current) charge/discharge processes, as shown in Fig. 1(c) and (d), were used to control the insertion and removal of Li ions into a HP film and thus the doping concentration continuously between 0% and 6% Li : Pb mol%. Blue lines show the electrochemical discharge (Li insertion/doping) processes and red lines the electrochemical charge (Li removal/“undoping”) processes, respectively (NB this colour convention is maintained throughout the manuscript). The current density applied *via* the potentiostat at each stage is shown in orange in Fig. 1(c). In order to measure the effects of Li insertion in the HP, a stable perovskite/electrolyte interface is necessary. Previous studies using battery-related technologies with HPs adopted the conventionally used liquid electrolytes, comprising Li salts in organic aprotic solvents. However, the polar solvents are prone to dissolving the HP at the electrolyte interface. The use of high molarity (>5 M) electrolytes reduced this effect and allowed global electrochemical measurements to be made in previous work.^30^ However, in order to undertake the detailed analysis shown herein, an intrinsically stable interface between the HP and electrolyte is required. As such, a polymer-based solid state electrolyte is used (LiTFSI in polyethylene oxide (PEO)). The use of this electrolyte results in an improvement in the stability of the HP/electrolyte interface of ∼82% over 24 hours compared to the conventional liquid electrolytes used in LIBs, calculated from the relative capacity retention (*cf.* ESI,† Section I).

**Fig. 2:**
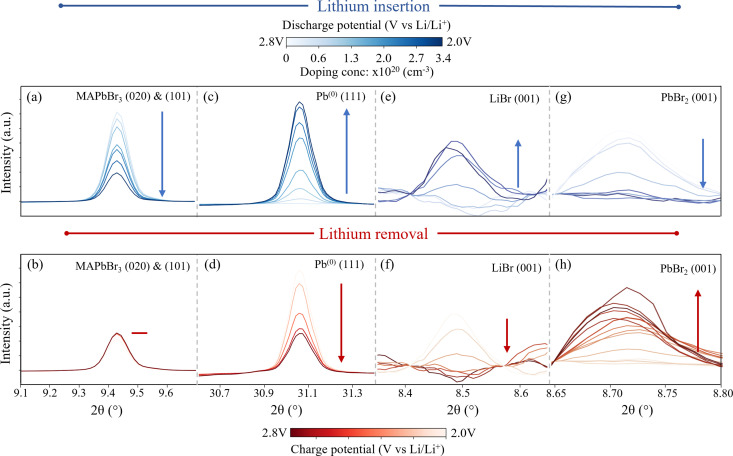
*Operando* XRD peak behaviour of each species rising and falling during the Li doping (blue) and removal (red) in MAPbBr_3_. (Wavelength *λ* = 0.9918 Å, 12.5 keV) Blue data corresponds to peak changes during Li insertion and red data to Li removal. Peaks proceed from low to high colour intensity as indicated by arrow. Peaks correspond to: (a and b) (020) and (101) MAPbBr_3_ (cubic space group *Pm*3̄*m*). The two orientations overlap and resolution is not sufficient to distinguish. (c and d) (111) Pb^(0)^ (cubic space group *Fm*3̄*m*). (e and f) (001) LiBr (hexagonal space group *P*6_3_*mc*) (g and h) Behaviour of the (001) reflection of PbBr_2_ during the first charge cycle (bottom red) and second lithiation (top blue) cycle. (orthorhombic space group *Pnma.*) The corresponding cell potential and doping concentrations for each peak are provided in colour bars at the top and bottom of the figure.

To elucidate the underlying mechanisms arising from the insertion of Li into MAPbBr_3_ and frame them in a quantifiable manner, *operando* XRD analysis was used during three successive Li ion insertion/removal cycles. Fig. 1(d) shows the three cycles accordingly, traversing the potential plateau at 2.2 V (insertion) and 2.4 V (removal), while Fig. 2 shows the response of various species observed during the first cycle. The top row shown in blue corresponds to lithium insertion increasing in concentration from 0 cm^−3^ to 3.4 × 10^20^ cm^−3^. The final doping state achieved after the first insertion step (1.8 V *vs.* Li/Li^+^) corresponds to a ratio of Li : Pb of ∼7%, *i.e.* (Li)_0.07_(MAPbBr_3_). A full conversion between doping concentrations and compositional ratios is provided in ESI,† Section II. The bottom row of Fig. 2 (red data) corresponds to the subsequent Li removal and clearly shows different degrees of reversibility for the species tracked in this XRD study. (Motion gif image files are provided in the ESI† that illustrate this reversible, oscillatory behaviour of the peaks clearly).

The first species investigated is the MAPbBr_3_ itself and the behaviour of the XRD fingerprint is demonstrated in Fig. 2(a) and (b) which corresponds to the (020) and (101) reflections that overlap at 9.45° 2*θ* (wavelength *λ* = 0.9918 Å, 12.5 keV). The behaviour of this peak is representative of all indexed perovskite peaks (additional peaks provided in ESI,† Section III) and proceeds as follows. Initially, as Li is inserted up to a doping concentration of 1 × 10^20^ cm^−3^ (corresponding to a molar doping concentration of 2% Li : Pb) the peak intensity remains relatively stable – dropping by only 3%. This corresponds to the initial lithiation process,

1 MAPbBr_3_ + *x*Li + *x*e^−^ → Li_*x*_:MAPbBr_3_

where *x* = <0.02. It is within this regime that we investigated the direct charge doping effects of lithiation in previous work by studying the Burstein–Moss shift as electrons are added into the conduction band of the perovskite.^55^

Following this initial insertion and continuing to a final doping concentration of 3.4 × 10^20^ cm^−3^ (Li_0.07_:MAPbBr_3_), the peak decreases in integrated intensity. The loss indicates that the inserted Li ions distort the perovskite structure, to the point where lattice order is reduced substantially in the material. As such, it is concluded that the prominent mechanisms are those that dramatically change the structure, including amorphization and conversions to non-perovskite compounds, rather than subtle modifications to the perovskite lattice structure, which may be expected if the Li ions were simply to sit interstitially within the lattice. It becomes clear, when the peaks that rise in place of the HP are studied, that the dominant process is indeed a conversion of the perovskite phase. A small diffraction signal attributable to HP is still observed after two subsequent Li insertion and removal processes, indicating that at these dopant concentrations, a small amount of the perovskite structure remains present. From this we also infer that performing multiple insertion and removal cycles results in a similar loss in diffraction peak intensity – indicating that it is not only the static doping concentration that causes the disruption of the perovskite crystal structure, but the very process of moving the lithium in and out. For the purpose of device modification therefore, we recommend the use of only a single insertion process when preparing the HP material for applications.

Next, we study the peaks that emerge as the perovskite peak intensity reduces. First, Fig. 2(c) and (d) show the behaviour of the (111) peak belonging to metallic lead (indexed phase: cubic *Fm*3̄*m*) during the doping (c) and subsequent lithium removal (d) processes. (All lead peaks behave in this manner, as shown in ESI,† Section III.) The peak area increases from zero (in the fresh perovskite sample) to a maximum during the first lithium insertion process, involving the insertion of Li ions to a doping concentration of 3.5 × 10^20^ cm^−3^. In addition to the single peak fitting of Fig. 2, Rietveld refinements of the time series spectra are used to extract approximations to the relative phase fraction between the MAPbBr_3_ and Pb^(0)^ at different doping concentrations. In doing so, we establish a relative phase fraction of perovskite:Pb^(0)^|0.3 : 0.7 at the end of the first insertion cycle. (*cf.* ESI,† Section IV for full details of the Rietveld refinement process). The formation of metallic lead during the discharge process is in accordance with ref. 25, 28 and 49. However, the reduction of Pb^2+^ to Pb^(0)^ is typically reported to occur at potentials below 1.4 V *vs.* Li/Li^+^.^58,59^ In this work however, the onset of the reduction of Pb^2+^ clearly occurs during the first galvanostatic plateau at ∼2.2 V *vs.* Li/Li^+^. This seemingly early onset could be attributed to the different local Pb environment in a HP compared to, for example, a lead metal oxide, which exhibits the same reaction, Pb^2+^ → Pb^(0)^ at 1.6 V *vs.* Li/Li^+^.^60^ Furthermore, previous studies mention the difficulty of detecting the exact onset of this reaction during *ex situ* studies – due to the propensity of the Pb^2+^ to react with ambient oxygen to form PbO before measurement.^58^ This effect is mitigated in this work by performing all measurements *in situ*, without exposing the electrodes to ambient oxygen. The use of a polymer electrolyte could also contribute to extending the overpotential of the reaction, shifting it to higher potentials than those reported previously.

The subsequent lithium removal cycle shown in Fig. 2(d), shows how the peak attributed to the metallic lead species drops accordingly, although not totally to zero. This result has two implications; first, the formation of metallic lead is partially irreversible in each cycle – resulting in a continuously increasing amount of the lead species with the number of cycles and the quanta of lithium doping. This is confirmed by phase fraction calculations extracted *via* Rietveld refinement (*cf.* ESI,† Section IV). These show a 100% Pb phase fraction relative to the perovskite after all three cycles are complete and the HP electrode left in the lithiated state. Second, since the XRD peak intensities of the perovskite itself decreases consistently with time, the general conversion of perovskite to lead is irreversible. This in turn has two concomitant and practical implications; first, that if one desires to use perovskites in an LIB device, for the purpose of energy storage, the irreversible nature of this first electrochemical process currently renders the reversible capacity of such a device to be extremely limited. It was possible to remove only 65% of the inserted Li ions from the structure during the first charge process, indicating that ∼35% is irreversibly locked up within the now formed species. This corresponds, in electrochemical terms, to a measured first cycle coulombic efficiency of 65% during the first cycle. Second, the insertion of excessive Li ions into a HP material causes initially the breakdown of the structure, including the reduction of the Pb^2+^ cation to Pb^(0)^.

In addition to the formation of Pb^(0)^, the growth of peaks corresponding to the species LiBr (indexed phase: hexagonal *P*6_3_*mc*) are observed during the Li insertion process as shown in Fig. 2(e). This is in accordance with Dawson *et al.*^57^ who propose the following conversion pathway of the perovskite during electrochemical cycling:

2 MAPbBr_3_ + 2Li^+^ + 2e^−^ → MABr + 2LiBr + Pb^(0)^

which describes the conversion of the perovskite into CH_3_NH_3_Br, LiBr and Pb^(0)^ and was derived from density functional theory showing how such a conversion, although not detected experimentally at the time, is energetically favourable. The fact that the products were not detected (other than Pb^(0)^, which was confirmed by *ex situ* XRD) was attributed to using a liquid electrolyte, in which the products are soluble and therefore not detectable using diffraction techniques. However, the use of the solid state electrolyte in this work now allows for them to be observed. The importance of such redox chemistry, particularly regarding the Pb^2+^ cation in the perovskite has been studied extensively for its relevance in the degradation of optoelectronic devices. For example, in the work of Zhao *et al.*,^61^ the Pb^2+^ cation is shown to be reduced by the interfacial reaction between the perovskite MAPbI_3_ and the Al electrode used in the device.

**Fig. 3:**
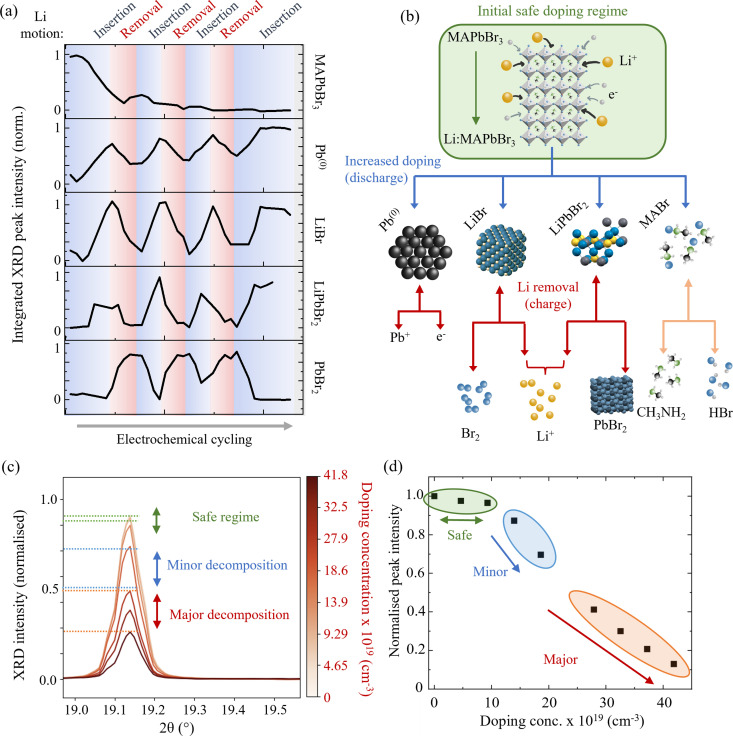
Li doping induced decomposition of MAPbBr_3_. (a) Normalised XRD peak integrated intensity as a function of multiple Li insertion and removal cycles for each of the studied species. Blue regions correspond to Li insertion and red to Li removal. (b) Schematic representation of the MAPbBr_3_ phase conversion pathways and decomposition products. Blue arrows denote processes that occur during Li insertion and red (downwards-pointing) arrows during Li removal. Decomposition of MABr (orange) is assumed to occur regardless of applied current.^62^ Double headed arrows denote bidirectional processes. (c) Close examination of Li insertion effects on MAPbBr_3_. Peak corresponds to the (032) (main peak) and (040) (left hand shoulder) of MAPbBr_3_ and is representative of all indexed MAPbBr_3_ peaks during this process. (Wavelength *λ* = 0.9918 Å, 12.5 keV.) (d) Integrated peak intensity as a function of doping concentration. Green, blue and red regions correspond to three doping regimes: safe, minor decomposition and major decomposition respectively, which are also indicated by horizontal lines on the diffraction peak in (c). Beyond the safe doping regime, a rapid decrease in perovskite peak intensity is observed.

Fig. 2(g) and (h) show the behaviour of the (001) peak attributed to PbBr_2_ (indexed phase: orthorhombic *Pnma*) during the first delithiation and subsequent second lithiation cycle. No peaks corresponding to this species are present in the fresh electrode and do not appear until the first Li removal cycle, indicating that this species is in fact formed from the perovskite conversion products. Once formed however, it reliably oscillates out of phase with the Pb^(0)^ and LiBr species, indicating a reversible shuttling between these two aggregate states. This behaviour is summarised in Fig. 3(a) where the normalized integrated peak intensity of all species is compared during the complete three-cycle electrochemical regime. By combining the experimentally observed behaviour of these species, we determine that in addition to eqn (1), the following process takes place during the Li insertion:

3 MAPbBr_3_ + Li + e^−^ → MABr + LiPbBr_2_

which describes the phase separation of the perovskite, with the intercalation of Li into the PbBr_2_ layers, forming LiPbBr_2_ and corresponding release of the MABr. Similar phase separation reactions into PbX_2_ and MAX have been shown for perovskites in the presence of oxygen and water^35^ albeit in the absence of Li.

Following the processes described by eqn (1)–(3) during Li insertion, the following processes proceed during the subsequent removal of the Li from the electrode, based on the response of the observed species’ XRD signals:

4 LiPbBr_2_ → Li^+^ + e^−^ + PbBr_2_

where eqn (4) describes the deintercalation of the Li from the PbBr_2_ structure, resulting in the appearance of the peak shown in Fig. 2(h): PbBr_2_, indexed phase orthorhombic *Pnma*. The liberated Li^+^ and e^−^ thence contribute to the apparent charging of the overall battery device when returned to the Li metal anode, but must not be mistaken for the reversible “recharging” of the perovskite, which by way of the conversion is irretrievably lost. Two distinct plateaus are observed in the charge cycle of the electrode at 2.3 V and 2.5 V (*vs.* Li/Li^+^) (*cf.*Fig. 1(d)). The second process at 2.5 V could include the stripping process of the now-formed Pb^(0)^ described by eqn (5):

5 Pb^(0)^ → e^−^ + Pb^+^

which has a standard electrode potential of −0.126 (aq) (2.9 V *vs*. Li/Li^+^). The potential offset observed here could be a result of the way that the pouch cell is assembled and cycled. For example, due to the heating of the cells to 70 °C in order to melt the PEO polymer. A contribution is also likely from the different electrolyte systems, PEO and LiTFSI compared to aqueous. This lead stripping process likely precedes the formation of PbBr_2_ and may explain why the increase in PbBr_2_ peak area occurs only in the second half of the removal process, lagging behind the drop in Pb^(0)^ intensity, as clearly observed in the trace in Fig. 3(a).

The integrated peak intensity for LiBr is observed to drop during Li removal cycles, which leads us to conclude that it likely decomposes chemically into its elemental constituent Li and Br_2_ by:

6 2LiBr ↔ 2Li + Br_2_

which has been previously demonstrated in the presence of H_2_O at voltages above +2.5 V *vs*. Li/Li^+^.^63^ Furthermore, after prolonged cycling – the pouch cell exhibits bubble pockets, as though gaseous species did indeed form during the cycling. Finally, we do not rule out the inevitable chemical decomposition of the MABr species formed during the Li insertion into its constituent HBr and CH_3_NH_2_, also gaseous, by:

7 MABr ↔ HBr + CH_3_NH_2_

a degradation mechanism studied at length in MAPbBr_3_ solar cell devices.^64,65^ A pictorial representation of the decomposition process resulting from the doping of MAPbBr_3_ is provided in Fig. 3(b) by the blue arrows. The subsequent compositional changes resulting from the “undoping”, or more accurately removal of Li are shown by the red arrows (reversible processes are denoted by double headed arrows and irreversible by single).

Having determined the implications of excessive Li doping in MAPbBr_3_, we now focus on smaller doping concentrations (<10^20^ cm^−3^), which are more typical and relevant for perovskite optoelectronic device applications. A closer examination of Fig. 2(a) and the integrated intensity trace of MAPbBr_3_ in Fig. 3(a) shows that the drop in perovskite diffraction signal does not proceed significantly until a doping concentration of ∼1 × 10^20^ cm^−3^ (corresponding to an elemental ratio of Li : Pb of 2%) is reached during the first insertion process. Fig. 3(c) shows this in greater detail where we break down the initial doping process into three regions: (1) safe, shown in green, (2) minor decomposition, shown in blue and (3) major decomposition, shown in red. Doping concentrations of up to ∼1 × 10^20^ cm^−3^ (corresponding to a Li : Pb ratio of 2%) are within the “safe” regime, whereby the crystal structure of the perovskite is little perturbed. At this doping concentration, we observe a drop in the integrated XRD peak intensity for the perovskite species of only 3% – measurements of which are shown in the green highlighted region of Fig. 3(d). We anticipate that this provides an experimental ceiling for any attempt at extrinsically doping a hybrid perovskite material. *In situ* doping methods, such as the demonstrated electrochemical doping, pushed beyond this limit are likely to initiate the decomposition of the perovskite material and therefore compromise the desired optoelectronic properties. Doping methods that take place *ex situ*, during the synthesis of the perovskite, by adding dopant salts to the precursor solution for example, are unlikely to result in the successful crystallisation of a single phase perovskite. For example, the work of Phung *et al.*^66^ shows how above doping concentrations of 0.1–1.0 mol% the chemical doping of Sr^2+^ or Mg^2+^ results in a surface phase segregation. Given that the ionic radii of the Mg^2+^ in the work of Phung *et al.* and the Li^+^ in our study are comparable (72 pm and 76 pm respectively), we propose that the fact that it is possible to insert twice as much Li^+^ is not due to geometric or volume based effects alone. That is, the phase segregation is not driven simply by the amount of space required for the dopants in the perovskite lattice. By considering the relative charge densities of the two ions, we conclude that the phase conversion/segregation is driven by a finite amount of charge density that the perovskite lattice can accommodate. Therefore, since the Mg^2+^ carries twice as much charge density as the Li^+^ – it follows logically that the ionic perovskite structure can only accommodate half as much Mg^2+^ as Li^+^ before it begins to decompose.^66^

Above this safe threshold of ∼2%, the peak intensity of the perovskite phase decreases rapidly with increasing lithiation. The effect is exemplified by the (032) and (040) reflection in Fig. 3(c) between the blue lines. Additionally, the effect on the total perovskite peak intensity with state of lithium doping concentration is shown in the blue highlighted area in Fig. 3(d). This region is denoted the minor decomposition regime between 1 × 10^20^ cm^−3^ (Li : Pb 2%) and 2.5 × 10^20^ cm^−3^ (Li : Pb 6%). At these doping concentrations, the perovskite phase remains the dominant phase (>50% calculated relative phase fraction compared to the products outlined in eqn (2) and (3)) but decreases sharply. The further addition of Li doping engenders significant drops in the perovskite diffraction signal and relative phase (from 98% to 50% phase fraction). This regime should be avoided when considering electrochemical doping as a method of doping perovskite materials for applications that require high levels of optoelectronic performance. (The modification to the optical properties will be considered in the relevant section below.) However, we note that the doping concentrations reached here, are already higher than those usually attempted for device applications, which are typically of the order of 10^18^ cm^−3^. The onset of this regime appears sharply at ∼2% Li : Pb mol%. Owing to the “soft” crystal structure characteristic of the HP class of materials,^67^ rather than decomposing linearly with added external species, the structure can withstand external species up to a specific loading threshold before suddenly dropping.

Finally, above these doping concentrations (from 2.5 × 10^20^ cm^−3^ to 4.5 × 10^20^ cm^−3^, or 6% to 9% Li : Pb ratio) the regime in which the perovskite no longer comprises the dominant species by relative phase fraction (<50%) is referred to as the majority decomposition regime. In this regime, the conversion processes, described by eqn (2) and (3), proceed so far that the signal from the perovskite decreases to <10% of its original intensity. Furthermore, Rietveld refinement of the spectra at this stage calculates a relative phase fraction between the perovskite and now formed lead to be 0.3 : 0.7 (ESI,† Fig. S6). We strongly caveat that in this regime the use of the word “doping” is no longer accurate since the onset of the conversion reactions in the previous regime demonstrate how the perovskite is now being converted to separate phases rather than being doped with interstitial lithium species. The (032) and (040) peak is shown to drop accordingly between the red lines in Fig. 3(c) and the overall effect on the perovskite reflection intensity is shown in the highlighted orange region in Fig. 3(d).

**Fig. 4:**
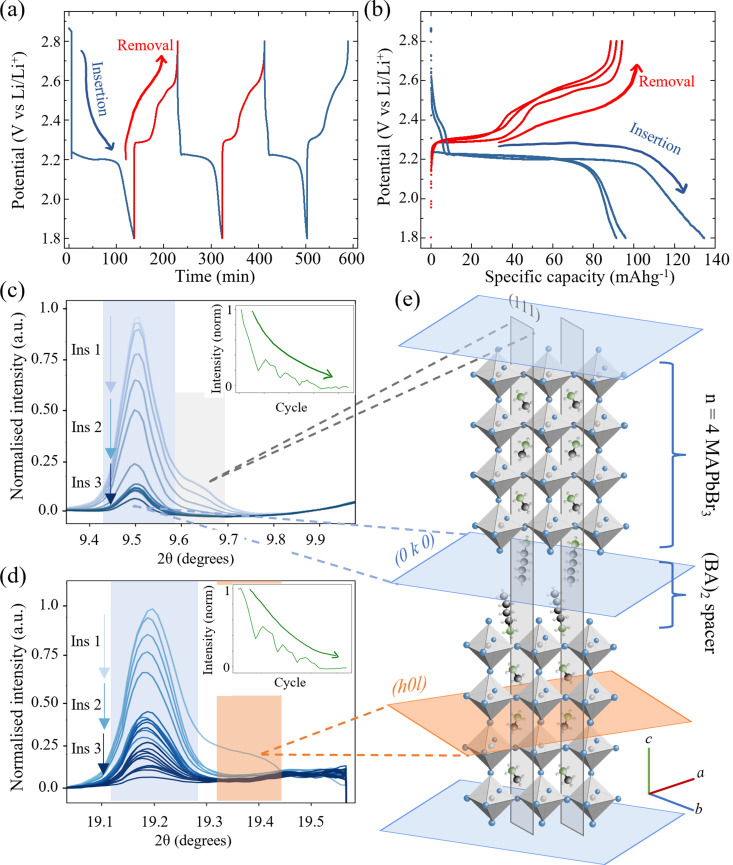
*Operando* XRD characterisation of 2d/3d hybrid perovskite (BA)_2_(MA)_3_Pb_4_Br_13_ during electrochemical doping. (a) Galvanostatic cycling protocol comprising three charge–discharge cycles at current density 60 mA g^−1^. (b) Corresponding charge–discharge voltage plateaus showing the electrochemical processes between 1.8 V and 2.8 V *vs*. Li/Li^+^ and their associated gravimetric capacities. (c) Behaviour of the (0 10 0) (blue) and (111) (grey) diffraction peaks of (BA)_2_(MA)_3_Pb_4_Br_13_ (indexed phase cubic *Cc*2*m*). Inset, normalised intensity of combined peaks during three insertion/removal cycles. (d) (0 20 0) (blue) and (202) (orange) reflections of the HP. (e) Schematic illustration of the crystallographic planes in (BA)_2_(MA)_3_Pb_4_Br_13_ showing the intra-perovskite reflections (grey and orange) and inter-perovskite reflections (blue). *N. B.* Planes are colour coded to match the respective peaks in (c) and (d). (Wavelength *λ* = 0.9918 Å, 12.5 keV.)

The *n* = 4 stoichiometric arrangement of the Ruddlesden Popper perovskite phase for the layered perovskites (BA)_2_(MA)_3_Pb_4_Br_13_ was investigated as an intermediate phase between the bulk-like perovskite MAPbBr_3_ discussed thus far and the quasi-2D (*n* = 1) phase^34^ (BA)_2_PbBr_4_ to establish generality for the HP decomposition reactions. The species can be considered as comprising four layers of MAPbBr_3_ intercalated between two longer BA cations. The galvanostatic cycling protocol was the same as for the MAPbBr_3_ above and comprised three discharge/charge cycles at a constant current of 1.5 μA (corresponding to 60 mA g_HP_^−1^). The corresponding potential/time and potential/capacity plots are shown in Fig. 4(a) and (b) respectively. Two regions of the diffractogram are shown in Fig. 4(c) and (d) that exhibit contributions from both intra- and inter-layer crystallographic planes. In both cases, the intra-MAPbBr_3_ layers (111) in (c) and (202) in (d) decrease in intensity rapidly during the first cycle shown and then remain negligible with subsequent cycles. This indicates that the same phase transformation processes of eqn (2) and (3) for the bulk perovskite hold in the quasi 2d/3d structure, with the decomposition of the MAPbBr_3_ layer occurring within the *n* = 4 perovskite layers. Interestingly, the inter-layer peaks (0 10 0) in (c) and (0 20 0) in (d) decrease in intensity much slower than the intra-layer peaks and are still observable after three complete cycles. Furthermore, the peak intensity recovers slightly (by ∼10% of relative intensity) during the charge (Li removal) processes. This is more evident when observing the combined normalised intensity shown inset in both (c) and (d) figures. The trace demonstrates a decreasing curve – representing the overall loss of the perovskite species with Li insertion and multiple cycles; however, with the superposition of an oscillatory signal caused by the partial recovery of the interlayer peak in each. This could indicate that despite the conversion of the MA_3_Pb_4_Br_13_ perovskite layers within the structure, that the inter-layer BA cation – being itself electrochemically inactive in this potential range between 1.8 V and 2.8 V *vs.* Li/Li^+^ – does not contribute electrochemically and does not host any inserted Li ions and thus does not exhibit such dramatic changes in the diffraction spectra due to deformation or conversion.

Since the crystal structure of the perovskite is one of the properties underpinning the optoelectronic character of the HP, the photoluminescence (PL) emission of the material is measured *in situ* during repeated Li ion insertion/removal cycles. (See ESI,† Section V for further experimental details.) First, one single insertion process is used to insert Li ions to a doping concentration of 8 × 10^18^ cm^−3^ (0.16% Li : Pb) before 20 complete cycles are executed between 1.8 V and 2.8 V *vs*. Li/Li^+^, inserting and removing a similar order of magnitude of Li ions. Finally, the device is electrochemically discharged to 0.1 V *vs*. Li/Li^+^ – inserting the maximum amount of Li ions to test what happens to the material in this extreme limit and an equivalent doping concentration of ∼1.1 × 10^21^ cm^−3^ (20% Li : Pb) is reached.

**Fig. 5:**
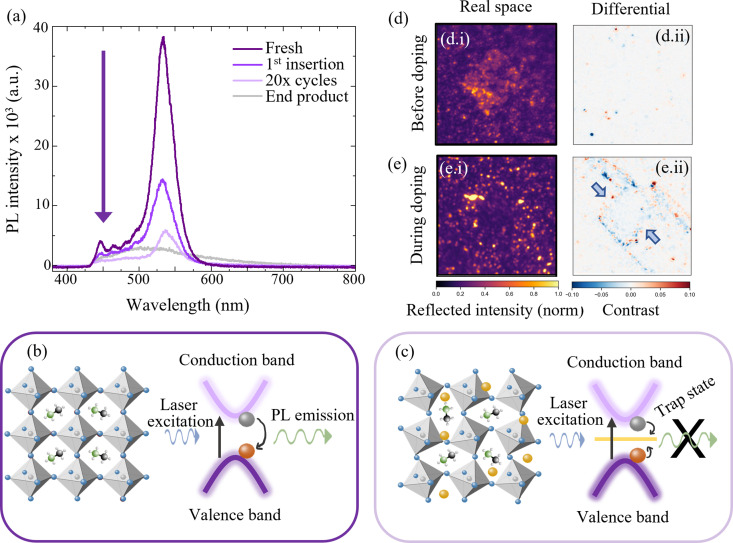
Optical characterisation of hybrid perovskites (BA)_2_(MA)_3_Pb_4_Br_13_ and MAPbBr_3_ during electrochemical doping. (a) Photoluminescence (PL) spectra of MAPbBr_3_ electrode at various stages of Li doping. (b) Schematic representation of the radiative band-to-band recombination of the unperturbed MAPbBr_3_ species. (c) Intercalation of lithium ions (yellow) and resulting lattice distortion leads to quenching of PL in the perturbed system. NB cartoon in (c) is approximate and does not represent a calculated structure. (d) (i) *Operando* reflection microscopy of (BA)_2_(MA)_3_Pb_4_Br_13_ before electrochemical insertion of Li showing a characteristic square crystallite. (ii) Differential frame analysis at *t* = 0 (before applying current). (e) (i) *Operando* reflection microscopy during electrochemical insertion of Li to an average (electrode-level) doping concentration of 3.0 × 10^20^ cm^−3^ showing loss of perovskite particulate structure. (ii) Differential frame analysis during the doping process (on the lithiation potential plateau at 2.1 V) showing inward procession of decreasing reflectivity. (Blue regions in differential plots correspond to loss in reflectivity and red to an increase in reflectivity.)

Fig. 5(a) shows the *in situ* PL characterisation of the MAPbBr_3_ electrode during these various stages of cyclic electrochemical doping. The characteristic emission of the perovskite species is used to infer the effect of lithium doping on the photoexcited charge carriers within the material and thus the changes to the energy structure. First, the strong emission shown in Fig. 5(a) in deep purple at ∼550 nm is characteristic of an unperturbed MAPbBr_3_ structure (*cf.*Fig. 5(b)). Upon electrochemical insertion of lithium ions to a representative doping concentration of 8 × 10^18^ cm^−3^ (0.16% Li : Pb), the PL emission intensity is observed to drop by ∼50% (1st insertion in Fig. 5(a)). Three concurrent effects contribute to this reduction in radiative emission. First, the introduction of trap-like energy states that lie within the bandgap of the perovskite semiconductor upon insertion of additional lithium species causes charges, that would otherwise recombine radiatively, to be trapped.^48^ Second, subtle changes to the perovskite phase crystal structure upon lithium doping are likely to affect the valence and conduction bands in position and curvature, diminishing the radiative recombination, while also allowing for additional lattice vibrations to couple to the optical excitations, resulting in increased non-radiative losses (Fig. 5(c)). Finally, the injection of one electron for every Li^+^ inserted in order to conserve charge neutrality results in an increase in the electronic doping concentration that is equal to the Li concentration. At the concentrations examined in this work of >10^17^ cm^−3^, the photoexcited holes will be rapidly quenched by the majority electrons thus reducing the charge carrier lifetime and PL emission.^55^ Given that we observe the preservation of >97% of the perovskite structure diffraction relative intensity at this level of doping, we can possibly infer that the main losses to the PL emission are not from the second mechanism described, but from the first and third – the introduction of trap states to the energy structure from the intercalated Li and from excited charge carrier quenching. This initial process is subsequently followed by twenty insertion and removal cycles, in order to determine the impact of repetitive cycling. As expected, the PL emission intensity drops due to the irreversible nature of the insertion and removal processes and the eventual conversion to non-emissive species, as described by eqn (1) and (2). The spectrum is labelled as “20× cycles” in Fig. 5(a) and shows a significantly reduced emission intensity from the perovskite species at 550 nm. Finally, for generality the cell is “deep cycled” whereby the electrode is fully lithiated by discharging to ∼0 V followed by a potential hold. This adds the maximum possible number of Li ions to the perovskite electrode and almost certainly results in additional side reactions, such as SEI formation, in addition to the phase conversions discussed before. The spectra from the cell after this step is labelled “end product” in Fig. 5(a) and shows a broad PL emission of very low intensity indicating a total loss of the emissive MAPbBr_3_ species.

*Operando* optical reflectivity (*cf.* ESI,† Section VI) was used to observe the changes to the 2d/3d perovskite (BA)_2_(MA)_3_Pb_4_Br_13_ directly and in real time with state of Li insertion.^68^Fig. 5(d) and (e) show reflection microscopy images obtained for a perovskite crystallite before and during the application of a constant current respectively. Subfigures (i) and (ii) refer in turn to reflected intensity images and differential frame images (see ESI,† Section VI for differential frame analysis method).^68^ Reflected intensity images show the raw reflected intensity of the perovskite crystallite and its surroundings and the differential images show the change in reflected intensity, at a given time, over a 40 s window. In this instance however, since the optical observations are limited to a localised region and indeed a single crystallite, we make no attempt to map the ensemble electrochemical state to that of the individually observed spot quantitatively. The ensemble electrochemical state (see ESI,† Fig. S10) is used as a guide as to where in the lithium insertion process the optically observable changes occur, without extrapolating to exact localised doping concentrations. Quantitative limitations aside, it is apparent that the square crystallite structure disappears as Li is added to the material from the electrochemical cycling (up to global Li concentrations of 3 × 10^20^ cm^−3^ (6% Li : Pb)). This implies that the crystallographic structure, responsible initially for the reflectivity image in Fig. 5(d)-i is changed beyond detection – as shown in Fig. 5(e)-i. This is attributed to the phase transformations described in eqn (2) and (3) as for the 3d MAPbBr_3_ analogue, since the products have neither the same cubic structure nor reflectivity spectrum as the initial perovskite.

The differential frame analysis is shown in Fig. 5(d)-ii and (e)-ii in which the dynamic changes occurring at the respective stationary frame emerge. Red regions correspond to areas of increasing reflectivity and blue areas to decreasing. An inwardly proceeding square of reflectivity loss (blue) is observed first at the edges of the crystallite before moving inwards. This confirms that the lithium ions originate from the electrolyte surrounding the crystallite and initially congregate at the outer edges. The inward motion corresponds to a homogenisation of the lithium ions through the perovskite structure, resulting in the initiation of the phase conversion reactions (eqn (2) and (3)) from the outside – in, finally resulting in the loss of the observable perovskite structure seen in (ei). For the reflectivity measurements, the material was discharged from OCP to 1.8 V *vs*. Li/Li^+^, with the current and time selected such that the final Li content was ∼6% Li : Pb – placing it on the border between the minor and major decomposition regimes described previously.

## 3. Conclusion

We utilise a solid state LIB-inspired device to investigate the effect of Lithium doping in the halide perovskite MAPbBr_3_ and the 2d/3d hybrid (BA)_2_(MA)_3_Pb_4_Br_13_ over a broad range of doping concentrations from 0 to >10^21^ cm^−3^ corresponding in compositional ratios of 0–20% Li : Pb. We demonstrate the use of a polyethylene oxide-based polymer as a stable electrolyte interface for HP materials that eliminates the dissolution effects encountered with liquid based electrolytes. This results in an improvement in relative capacity retention of 82% after 24 hours in electrode calendering stability.

We demonstrate the ability to dope HPs with a high degree of precision and perform a suit of *operando* analyses of the material to study structural changes of HPs during doping. Synchrotron XRD measurements quantify three doping regimes; a safe regime, below doping concentrations of ∼10^20^ cm^−3^ (0–2% Li : Pb mol%) in which the HP may be doped without detrimental effects to its structure; a minor decomposition regime between doping concentrations of 10^20^ cm^−3^ and 2.5 × 10^20^ cm^−3^ (Li : Pb ratio of 2% to 6% respectively), in which the HP phase is partially transformed but remains the dominant species. Finally, we quantify a major decomposition regime (>2.5 × 10^20^ cm^−3^ corresponding to Li_*n*_:HP where *n* > 0.06) in which the perovskite is superseded in relative phase fraction by new conversion products including Pb^(0)^, LiBr and LiPbBr_2_. By comparison to previous studies which use Mg^2+^ as the dopant species,^66^ we conclude that the dopant charge density is as important to stabilising the HP as the ionic radius of the species – explaining why twice as much Li^+^ as Mg^2+^ may be incorporated into the ionic structure. The results are confirmed by *in situ* photoluminescence spectroscopy which isolates the charge carrier doping effect from structural effects. *Operando* optical reflection microscopy shows the real-space motion of the conversion processes. In doing so, we provide an experimentally derived, quantitative doping framework with which future devices reliant upon the doping of perovskite thin films may be based.

## 4. Experimental methods section

### 4.1. HP synthesis

Powders of the various perovskites studied in this report were prepared by using a slow evaporation technique. First, solutions of Ruddlesden–Popper hybrid perovskites were prepared by mixing stoichiometric amounts of the precursor powders in dimethylformamide (DMF) under an argon atmosphere. The precursors are listed in Table S2 (ESI†) for reference. The resulting solutions were stirred using a magnetic stirrer at 70 °C for 1 hour to ensure total and homogeneous dissolution. After cooling, the solution was transferred to a vacuum oven and dried overnight at 60 °C. The powder precipitate was collected and stored again under an argon atmosphere.

### 4.2. Electrode fabrication

Electrode fabrication was undertaken in an argon atmosphere with H_2_O and O_2_ < 0.5 ppm. The electrode slurry consisted of 85 wt% perovskite powder, 5 wt% polyvinylidene difluoride (PVDF, Arco Ltd) binder, and 10 wt% conductive carbon (Super-P, Alfa Aesar), and was produced stepwise, as follows. The perovskite powder was added to anhydrous *N*-methyl-2-pyrrolidone (NMP, Sigma Aldrich) into which it dispersed. The PVDF binder was then added and the solution stirred magnetically overnight to ensure homogeneity. Finally, the Super-P was added and the resulting solution was sonicated for 3 hours in an ultrasonic bath. The solution was then used to deposit perovskite electrodes by drop casting on a pre-cut circular (*ϕ* = 12 mm diameter) substrate of choice – depending on the specific cell architecture for the desired experimental setup. 6.3 μL of solution was dropped, corresponding to a mass loading of 0.36 mg cm^−2^ of active material. After drop casting, the as coated electrodes were annealed at 90 °C overnight (at least 12 h) to ensure complete removal of the NMP.

### 4.3. PEO LiTFSI solid state polymer electrolyte synthesis

First, the PEO (PEO10 : 600k molecular weight, Sigma Aldrich) was dried under vacuum at 80 °C and the LiTFSI salt under vacuum at 160 °C. 1.25 g of PEO, 816 mg of LiTFSI and 11 mL of acetonitrile were mixed together under an argon atmosphere (<0.5 ppm H_2_O, O_2_) and stirred for 24 hours at 360 rpm, using a magnetic stirrer. The solution was degassed by pumping the solution in an open container three times, using the small antechamber of the argon glovebox, in order to remove all of the bubbles in the solution. The solution was coated onto a sheet of Teflon™ using a doctor blade coater at room temperature, set to a thickness of 254 μm. The film was dried for 48 hours at 70 °C in the argon atmosphere using a hotplate. The protocol is based on that of ref. 69.

### 4.4. Electrochemical cell architectures

Various cell architectures were used to characterise the HP electrodes in the different measurement setups as follows:

#### 4.4.1. EL electrochemical cell for *operando* reflection microscopy

The prepared HP electrode solution was drop cast onto a glass cover slip and mounted into an optical cell (EL-CELL, ECC-Opto-Std test cell) as shown in ESI,† Fig. S9. A full description of the assembly process is provided in ESI,† Section VI. It is important to note that the polymer electrolyte is not compatible with the EL cell and therefore for the optical reflection microscopy measurements a high concentration electrolyte (5 M LiTFSI in EC : PC 1 : 1 mol ratio) was used. We show in previous work how the high salt however provides a window of stability long enough for these measurements to be undertaken.^30^

#### 4.4.2. Optical LIB coin cell device for *in situ* PL measurement

A custom-built coin cell with an optical window was used to measure the *in situ* PL emission of the perovskite material under various stages of electrochemical doping (schematic provided in ESI,† Fig. S8). Coin cells (CR2032) were modified by drilling a hole of diameter *d* = 8 mm into the bottom casing. The hole was sealed using a transparent glass window with EPOXY (EVO-STIK). Digital photographs of this cell type, in addition to an extended description of the fabrication are provided in ESI,† Section V. For this experiment, a pure perovskite solution was used (omitting the PVDF binder and Super-P conductive additive) in order not to interfere with the PL emission.

#### 4.4.3. Temperature controlled, high pressure pouch cell for *operando* X-ray measurements

A pouch cell architecture, in conjunction with a custom 3D printed holder was used to perform *operando* X-ray measurements. The as-prepared perovskite electrode slurry was drop cast on to indium tin oxide (ITO) coated polyethylene terephthalate (PET) and assembled in the pouch cell, as described in ESI,† Section VIII. The assembled pouch cell was mounted in a bespoke holder, which utilized a Kapton window for X-ray access. A high-pressure Kapton dome with a gas supply was used to apply a constant high pressure of 30 PSI through the cell^70^ and an electrical heating element used to raise the LiTFSI/PEO to the required temperature of 70 °C, where it is molten and conductive.

### 4.5. Optical reflection microscopy

Optical reflection microscopy was carried out by adapting a previously described microscope setup.^68^ A home-built inverted microscope equipped with an oil immersion objective (100×, UPLSAPO100XO, Olympus) and polarisation optics in the detection path imaged the reflected and scattered light from the sample onto a CMOS detector (FLIR, Grashopper3, GS3-U3-23S6M-C) with an overall magnification of 166.7×. In this work, the sample was illuminated at 740 nm by a high power LED source (Thorlabs SOLIS-740C), equipped with a ground glass diffuser to minimise speckle contributions and homogenise the illumination. The field of view was controlled by a field aperture and set to achieve a circular illumination profile with a diameter of 35 μm.

The optical cell (EL-CELL, ECC-Opto-Std test cell) was mounted on an XYZ nano-positioner stack (Attocube, ECSx3030/AL/RT/NUM) with an overall travel range of 25 mm in all dimensions. The sample focus position was maintained *via* an active external focus stabilisation based on a calibrated line-reflection profile of a 980 nm reference laser, as described previously.^68^ Comprehensive details of the experimental setup and data acquisition are explained in ESI,† Section VI.

### 4.6. *In situ* XRD characterisation

*In situ* XRD characterisation was undertaken at the beamline 10-2 at Stanford Synchrotron Radiation Light Source (SSRL) at SLAC National Laboratory (wavelength *λ* = 0.9918 Å, 12.5 keV). The beam size was defined by slits to 0.3 × 0.3 mm. Scattered photons were collected using a DECTRIS Pilatus 300 kW photon counting detector, placed 250 mm from the sample on a diffractometer arm. It was oriented in portrait mode to cover a large range of scattering angles and could be moved on the diffractometer arm to cover the desired scattering angles. The pouch cell assembly, described in Section 4.4.3 above was placed in the beam-line. 50 spots were defined over the area of the perovskite electrode and were exposed using a raster method for approximately 15 s per exposure to minimise beam damage to the sample. A total of 62 spectra were collected for each spot over the course of a full 4× insertion/removal cycling process (3100 spectra per sample). A full description of the synchrotron experimental setup and analysis are found in ESI,† Sections VIII and IX, respectively.

### 4.7. *In situ* photoluminescence spectroscopy

To measure the PL spectra of the perovskite at a given state of charge, the perovskite thin film was mounted in a customised optical coin cell, described in Section 4.4.2 above. Steady-state PL spectra were recorded by a gated intensified CCD camera (iCCD, Andor Star DH740 CCI-010) connected to a grating spectrometer (Andor SR303i). The pulsed output from a mode-locked Ti:Sapphire optical amplifier (Spectra-Physics Solstice, 1.55 eV photon energy, 80 fs pulse width, 1 kHz repetition rate) was used to produce 400 nm excitation *via* second harmonic generation in a β-barium borate crystal. The iCCD gate (width 2 ns) was electronically stepped in 2 ns increments, relative to the pump pulse, to enable ns-temporal resolution of the PL decay. A full description of the experimental setup is provided in ESI,† Section V.

## Conflicts of interest

There are no conflicts to declare.

## Supplementary Material

EE-015-D2EE01754G-s001

EE-015-D2EE01754G-s002

EE-015-D2EE01754G-s003
