# Supplementary material for: A mechanistic study of the dopant-induced breakdown in halide perovskites using solid state energy storage devices
Source: Energy Environ Sci. 2022 Sep 6;15(10):4323–37. doi: 10.1039/d2ee01754g (PMC9555316; doi:10.1039/d2ee01754g)
Supplement: EE-015-D2EE01754G-s003 [file EE-015-D2EE01754G-s003.pdf]

Electronic Supplementary Material (ESI) for Energy & Environmental Science.
This journal is © The Royal Society of Chemistry 2022

Supplementary Information

A Mechanistic Study of the Dopant-induced Breakdown in Halide Perovskites using Solid State Energy Storage Devices

Angus Mathieson[1,2,3], Wesley M. Dose[1, 4], Hans-Georg Steinrück[5,6], Christopher J. Takacs[6], Sascha Feldmann[2,7], Raj Pandya[2], Alice Merryweather[2], David Mackanic[6], Akshay Rao[2], Felix Deschler[2] & Michael De Volder[1]**

[1] *Institute for Manufacturing, Department of Engineering, University of Cambridge, 17 Charles Babbage Rd, Cambridge CB3 0FS*

[2]*Cavendish Laboratories, Department of Physics, University of Cambridge, 17 JJ Thomson Ave, Cambridge, CB3 0HE*

[3] *Cambridge Graphene Centre, Department of Engineering, University of Cambridge, 9 JJ Thomson Ave, Cambridge, CB3 0HE*

[4] *Department of Chemistry, University of Cambridge, Lensfield Road, Cambridge, CB2 1EW*

[5] *Department Chemie, Universität Paderborn, 33098 Paderborn, Germany*

[6] *SSRL Materials Science Division, SLAC National Accelerator Laboratory, Menlo Park, California 94025, United States*

[7] *Rowland Institute, Harvard University, Cambridge, Massachusetts 02142, United States*

*mfld2@cam.ac.uk, felix.deschler@tum.de

Section I: PEO solid state polymer electrolyte

Figure S1: Necessity of a solid state electrolyte compared to liquid alternatives. (a) Galvanostatic charge-discharge curves of cells containing a conventional liquid electrolyte (1M $LiPF_6$ in EC/DEC) left a defined time between assembly and cycling. A loss of cycle capacity is observed due to solvent-induced degradation of the perovskite material. Shaded regions highlight the characteristic voltage plateaus at 2.1 V and 1.5 V in the initial discharge cycles. Inset Zoomed axes of the second cycle onwards for each cell. **(b)** Digital photographs of a $MAPbI_3$ perovskite before and after contact with the liquid electrolyte showing immediate dissolution. **(c)** First galvanostatic discharge/charge curve for a PEO based perovskite battery cell, showing the increase in background stability from the polymer electrolyte.

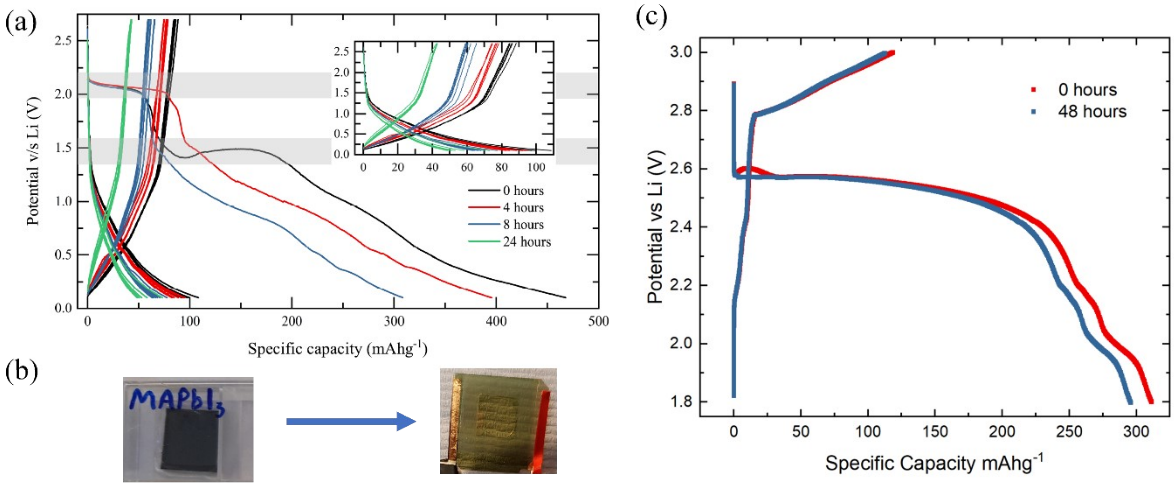

A polymer (polyethylene oxide (PEO))-based electrolyte was used throughout the work to eliminate the underlying degradation of the perovskite due to its inherent solubility in polar solvents, otherwise encountered when using conventional liquid-based electrolytes. **Fig. S1** demonstrates the

significance of the dissolution of the perovskite active material on the measurable electrochemical gravimetric capacity of a conventional lithium ion battery (LIB) using a traditional liquid electrolyte. (1M $LiPF_6$ in EC/DEC, 1:1 vol ratio). **Fig. S1(a)** shows the galvanostatic charge-discharge cycles of the cells, inset are the zoomed axes showing the second cycles onwards. The time indicated corresponds to the length of time left between assembling the cell and then beginning the initial discharge cycle.

All cells were cycled at the current rate of 30 mAg^{-1}. It is immediately apparent that both the initial discharge capacity and the stabilized capacity are reduced, the longer the cell is left before beginning the cycle test. The best performing cell in both the first and all subsequent cycles was the cell that was cycled immediately upon assembly (0 hours). The cells left for four and eight hours follow a similar drop in capacity. The most prominent change occurs between eight hours and twenty four hours. The cell that was left for twenty four hours before cycling observed a drop of ≈ 400 $mAhg^{-1}$ (~80%) compared to the other cells for the initial discharge capacity. Since it is proposed that the dominant processes responsible for large initial discharge capacity are the lithium insertion into and the subsequent conversion reaction of the perovskite structure, it may be hypothesized that the drop in capacity after twenty four hours is due to the absence of these two mechanisms - indeed, no plateaus are observed at 2.1V or 1.5V to contradict this. It could be concluded therefore that after twenty four hours of contact with the electrolyte, the perovskite structure has been dissolved - or at least compromised beyond the point of being able to facilitate lithium insertion or conversion. Traditionally (in non-perovskite cells) it is common to leave cells for a length of time, up to around twenty four hours before cycling in order to allow the electrolyte time to soak the electrode completely, increasing the coverage and thus active surface area.

The final plateaus, below 1.0 V are all approximately equal in capacity irrespective of the time taken between cell assembly and measurement. Since the reaction below 1.0 V is a multistage alloying reaction between lead and lithium - it is unsurprising that the magnitude of capacity contained within this reaction is equal for the samples which, being the same perovskite formulation, inherently contain the same quantity of lead - irrespective of the state of dissolution. For these experiments, a conventional coin cell was used.

A simple, ex situ stability test was carried out by dropping a sample of liquid 1M electrolyte onto a typical film of $MAPbI_3$ -the iodide analogue of the perovskite used in this work. Upon leaving the sample for 24 hours, the metallic-black characteristic colour and texture of the $MAPbI_3$ is totally lost and in its place is a yellow solution, as shown in **Fig. S1 (b)**. The remaining yellow solution is indicative of dissolved PbI_2 salts – one of the precursors to making $MAPbI_3$. Therefore, it can be concluded that in any battery cell, using liquid electrolytes containing polar solvents, that any OIHP material contained within the electrode will dissolve into its respective PBX_2 lead salt (where X denotes the relevant halide anion).

Finally, **Fig. S1(c)** shows the corresponding galvanostatic charge-discharge curve of a $MAPbBr_3$ electrode, in a conventional LIB coin cell, using however, the polymer-based electrolyte described herein. A small capacity decrease of ~30 $mAhg^{-1}$ (< 10%) after 48 hours is observed, smaller already than the loss observed in the liquid cell after only 4 hours. This indicates an immense improvement to the overall stability of the perovskite/PEO interface relative to the perovskite/liquid electrolyte.

Section II: Calculation of doping concentration and unit conversion charts

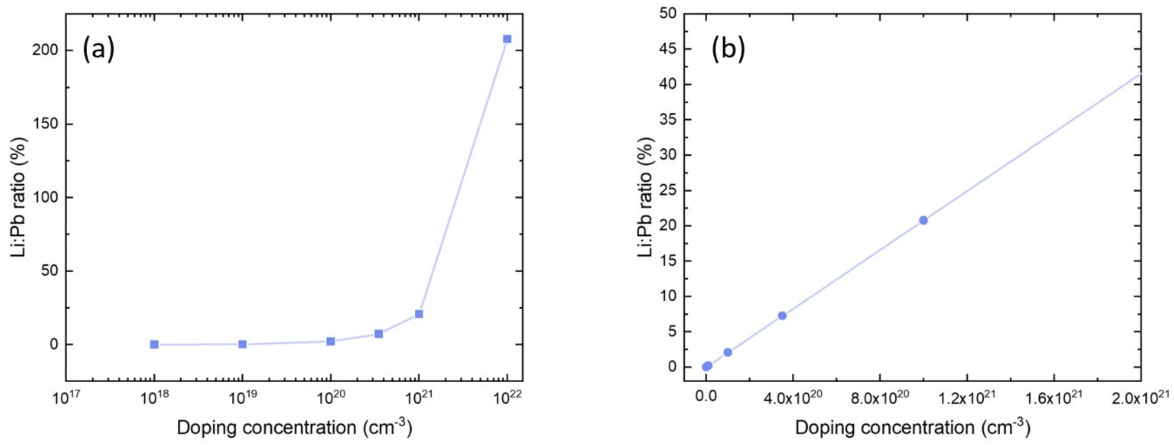

Fig. S2: Conversion between the units used interchangeably in the main text. Doping concentrations (x-axis) are used when appropriate for discussing optoelectronic devices and the corresponding molar ratio between inserted Li and native Pb shown on the y-axis.

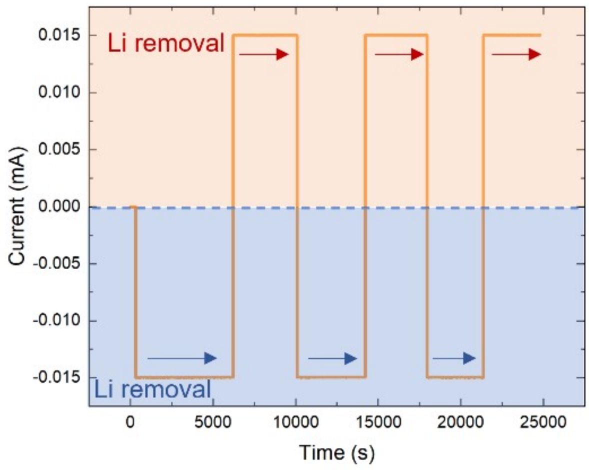

Fig. S3: Example current-time plot of the galvanostatic electrochemical process used to insert and remove Li ions from the perovskite material. The amount of lithium moved between the anode (Li metal) and cathode (perovskite) is determined by multiplying the measured current by the time for each step. Blue region (negative current) corresponds to the Li insertion processes and red region (positive current) to Li removal processes.

The number of lithium ions inserted into the perovskite material electrochemically and therefore the doping concentration is determined is calculated using the following:

The number of Li ions that are transferred from the anode (Li metal) to cathode (perovskite) are measured via the potentiostat as the equivalent number of electrons transfer through the external circuit and manifest as a constant current (I), as shown in **Fig. S3**.

The number of charges passed through the external circuit (Q) in Coulombs is simply calculated by,

$$Q = I \times t \tag{2}$$

Where t (seconds) is the time elapsed during a given current condition.

Since one Coulomb of charge corresponds to 6.24×10^{18} charges, the number of charges and thus Li ions transferred through the cell (N) is given by,

$$N = \frac{Q}{1.6 \times 10^{-19}} \tag{3}$$

where 1.6 x 10^{19} C is the charge of the electron. The doping concentration (conc.) is then calculated using the volume V (cm^3) of the perovskite electrode (in cm^{-3}) thus,

$$conc. = \frac{N}{V} \tag{4}$$

For ease of reference, at the used current rate of ± 0.015 mA where a negative corresponds to discharge and therefore Li^+/e^- insertion and a positive corresponds to charge and therefore Li^+/e^- removal, inserting the correct values yields a charge doping insertion or removal rate of 9.36 x 10^{13} Li/e^- per second.

To calculate the molar ration (Li:Pb %) the number of Pb species per unit volume (cm^{-3}) is calculated by assuming 1 Pb per perovskite unit cell, giving a value of 4.81 x 10^{20} Pb per cm^{-3}.

Section III: Additional peaks and GIFs showing the species behaviour in real time

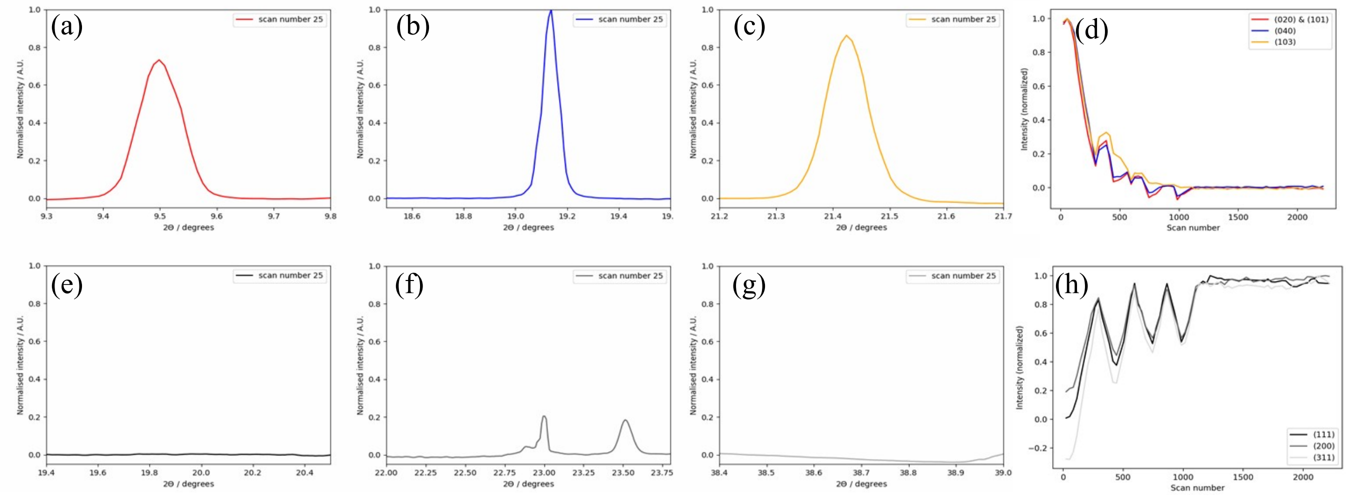

Fig. S4: Motion GIF images of additional peaks of $MAPbBr_3$ and $Pb^{(0)}$ showing universal responses: (a - d) Additional peaks associated with $MAPbBr_3$. (indexed phase: cubic *Pm-3m*). **(a)** (020) and (101). **(b)** (040) **(c)** (103) **(d)** Integrated peak area intensity for the three peaks with multiple insertion/removal Li cycles. All peaks unanimously decrease rapidly with increased Li insertion and cycling. **(e – f)** Additional peaks of metallic $Pb^{(0)}$. (indexed phase: cubic *Fm*-3*m)*. **(e)** (111) **(f)** (200) **(g)** (311) **(h)** Integrated peak area intensity for the three peaks with multiple insertion removal cycles. Peaks unanimously increase with Li insertion and decrease with Li removal. *NB: Motion pictures available for download from https://pubs.xxx as additional supplementary file.*

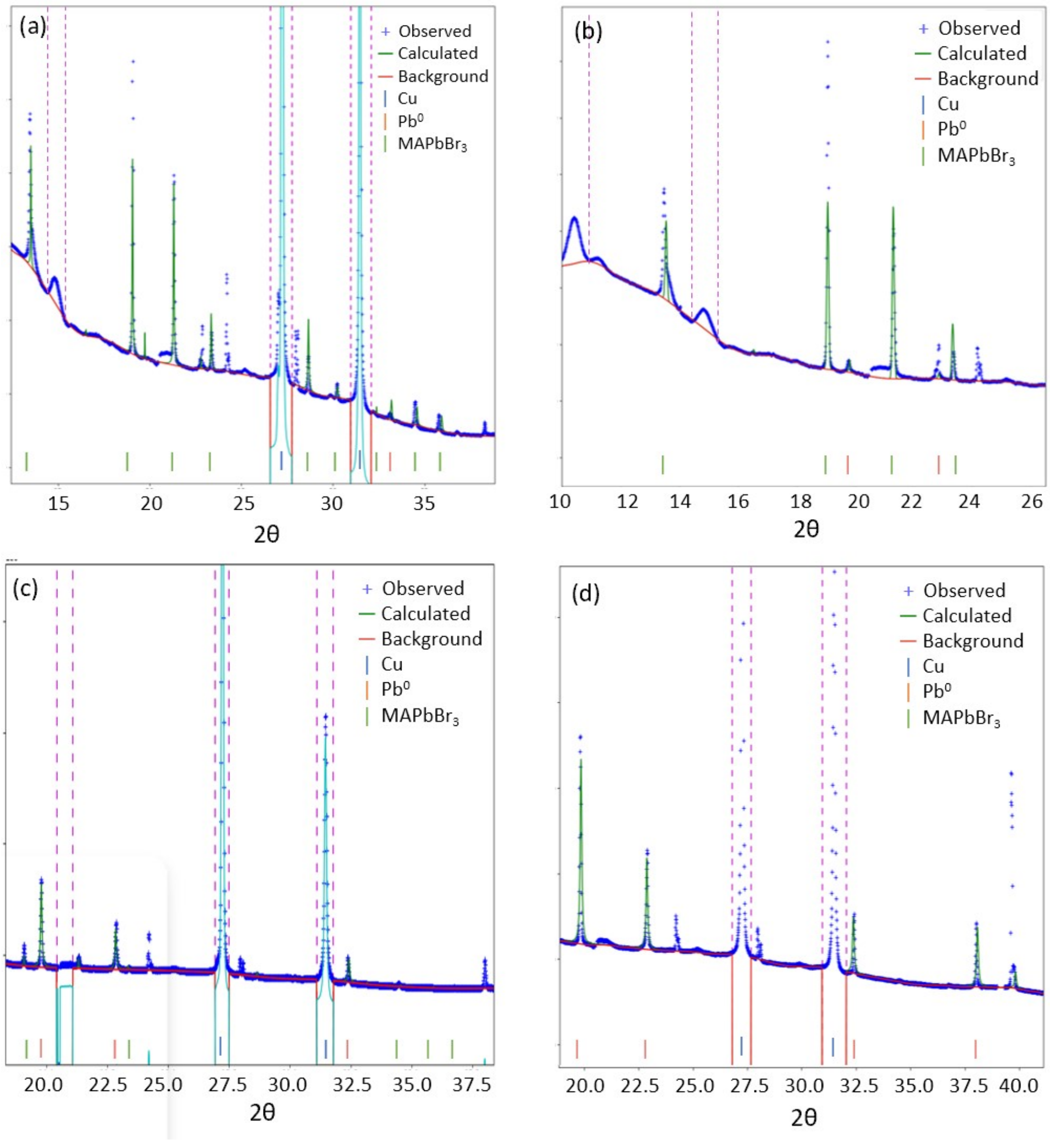

Fig S5: Rietveld refinement fitting for four XRD spectra taken at different stages of Li doping cycles. (a) First spectra taken at given position representing the material structure at the beginning of the first Li insertion cycle. Doping concentration of 0 cm^{-3} and compositional ratio of Li_0:$(MAPbBr_3)_{1.0}$ **(b)** Spectrum reference 85 after fitting. Both $MAPbBr_3$ and $Pb^{(0)}$ features exist and a relative phase fraction of perovskite:$Pb^{(0)}$ of 1:99 is calculated. Doping concertation at this stage of 7.47 x 10^{19} cm^{-3} and compositional ratio of $Li_{1.5}$:$(MAPbBr_3)_{98.5}$. **(c)** Spectrum reference 295 after fitting. Both $MAPbBr_3$ and $Pb^{(0)}$ features exist and a relative phase fraction of perovskite:$Pb^{(0)}$ of 24:76 is calculated. Doping concertation at this stage of 2.85 x 10^{20} cm^{-3} and compositional ratio of $Li_{0.6}$:$(MAPbBr_3)_{0.94}$. **(d)** Spectrum reference 1225 (after three complete Li insertion and removal cycles). Perovskite reflections are reduced beyond detection and are replaced by $Pb^{(0)}$ features of maximal intensity. Corresponds to a final doping concentration of 3.5 x 10^{20} cm^{-3} and a compositional ratio of $Li_{0.073}$:$(MAPbBr_3)_{0.927}$.

Section IV: Rietveld refinement

Rietveld refinement was used to fit the XRD spectra at four different doping stages. Spectra 25 – at the beginning of the first insertion process, spectra 85 – at a low doping concentration of 7.47 x 10^{19} cm^{-3} during the first insertion process, spectra 295 at a high doping concentration of 2.85 x 10^{20} cm^{-3} at the end of the first insertion process and spectra 1225 at a net doping concentration of 3.5 x 10^{20} cm^{-3} at the end of the third insertion process and therefore including two removal processes prior.

A preliminary refinement was used to approximate the spectra before then using a Le Bail extraction [1] to calculate better peak positions for the various components included in the fit. These parameters were then fed back into the Rietveld refinement to achieve the final fit. Due to artefacts introduced during the operando XRD experiment, a perfect refinement was not achievable – this is reflected in the final χ^2 values in Table (S1). The authors would therefore like to exercise caution with overreaching the parameters able to be extracted from the refinement reliably.

However, by limiting the refinement to a closed system comprising only the $Pb^{(0)}$ and $MAPbBr_3$ species, the relative phase fractions of these two – which are necessarily dependent upon the relative peak area intensities – the shift in ratio between these two present species was extracted with reasonable accuracy. Indeed, the results corroborate the single peak fitting described in the main text. The resulting relative phase fractions are shown in **Fig. S6 (c))**.

The main parameters used to refine the model include the unit cell length for each species, its μ-strain and domain size. For completeness these parameters and how they vary with each spectra are shown in **Fig. S6** and are tabulated in **Table S1**.

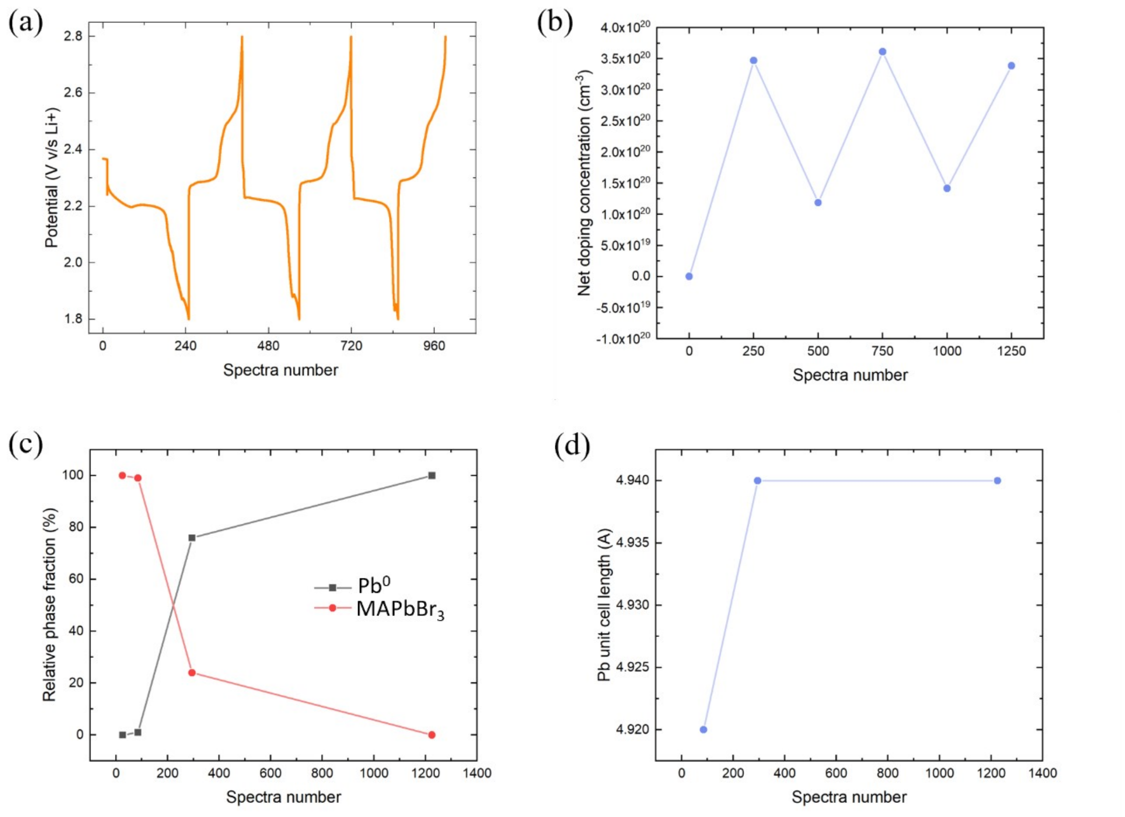

Fig S6: Rietveld refinement extracted parameters for Pb$^{(0)}$ and MAPbBr$_3$. (a) Spectra number vs electrochemical potential for the first three insertion and removal processes for reference to the extracted spectra below. **(b)** Net doping concentration (concentration remaining after each electrochemical step) for first three insertion cycles. **(c)** Relative phase fraction calculated for Pb$^{(0)}$ (black trace) and MAPbBr$_3$ (red trace) with time (c.f. (a) and (b) for conversion to potential and doping concentration.) The data highlights the transfer of the dominant species in the electrode from perovskite to lead during increased doping cycles. **(d)** Increase in the calculated unit cell length for Pb$^{(0)}$ during doping of the electrode.

Spectra number	25	85	295	1225
Cycle time (s)	~0	1588	7153	31,806
Doping concentration (cm^{-3})	~0	7.47×10^{19}	2.85×10^{20}	3.5×10^{20}
Li_x:$(MAPbBr_3)_y$	Li_0: $(MAPbBr_3)_{100}$	$Li_{1.5}$:$(MAPbBr_3)_{98.5}$	Li_6:$(MAPbBr_3)_{94}$	$Li_{7.3}$:$(MAPbBr_3)_{92.7}$
Pb^0 relative phase fraction (%)	0	1	76	1
Perovskite phase fraction (%)	100	99	24	0
Perovskite domain size (µm)	10	8	0.4	-
Perovskite unit cell length (Å)	5.88	5.91	5.88	-
Fitting χ^2	142	76	201	117

Table S1: Summary of extracted parameters from the Rietveld refinement of the $MAPbBr_3$ XRD spectra at various doping concentrations.

Section V: Photoluminescence measurements and Optical Coin Cell fabrication

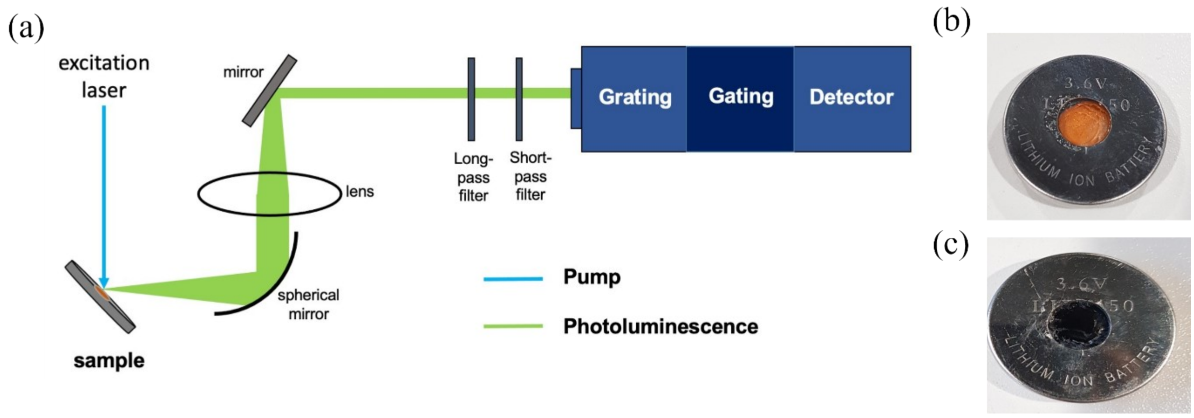

Fig. S7: (a) Schematic representation of the photoluminescence setup used to characterise the PL emission of the perovskite at various stages of Li doping and degredation. **(b)** Fresh $MAPbBr_3$ perovskite optical coin cell, used to measure the PL emission of the material prior to any Li insertion. **(c)** Final $MAPbBr_3$ optical coin cell, after multiple charge insertion and removal processes.

A schematic of the setup used to measure the in situ PL spectra of the perovskite material, under various stages of Li doping is shown in **Fig. S7 (a)**. Steady-state PL spectra were recorded by a gated intensified CCD camera (iCCD, Andor Star DH740 CCI-010) connected to a grating spectrometer (Andor SR303i). The pulsed output from a mode-locked Ti:Sapphire optical amplifier (Spectra-Physics Solstice, 1.55 eV photon energy, 80 fs pulse width, 1 kHz repetition rate) was used to produce 400 nm excitation via second harmonic generation in a β-barium borate crystal. The iCCD gate (width 2 ns) was electronically stepped in 2 ns increments, relative to the pump pulse, to enable ns-temporal resolution of the PL decay.

A digital photograph of a fresh $MAPbBr_3$ optical coin cell, prior to any electrochemical processing is shown in **Fig. S7 (b)**. The orange colour is characteristic of the starting perovskite

material. The same cell, after multiple deep insertion and removal processes is shown in **Fig S7 (c)**. The black colouration is characteristic of the final degredation products after multiple cycles, predominantly metallic lead.

The internal stacking of the optical coin cell is similar to that of the pouch cell described in **Section (IX)**. First, the perovskite material is drop cast onto an FTO coated piece of glass and annealed at 70 °C for 24 hours in an argon glovebox (<0.5 ppm H_2O and <0.5 ppm O_2). Copper contacts are used to connect the FTO to the internal surface of the stainless steel coin cell, which has a hole of diameter d = 8 mm drilled into the centre. The rest of the cell is assembled by placing a glass fibre separator, a layer of PEO polymer electrolyte, metallic lithium anode and stainless steel spacer and spring on top. The cell is sealed using a crimping machine at 1000 PSI.

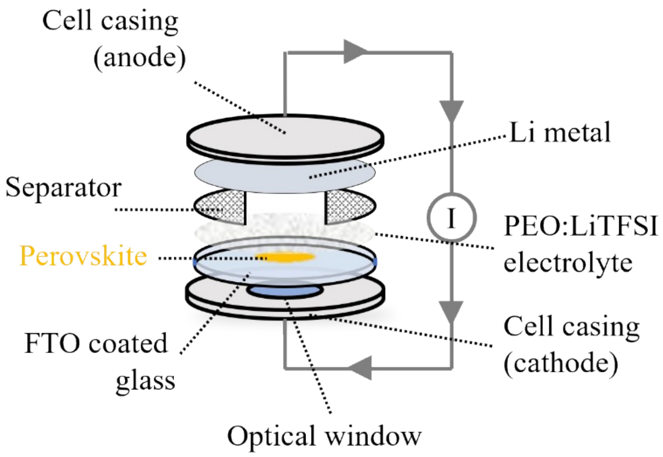

Fig. S8: Schematic illustration of the bespoke optical coin cell used for in situ photoluminescence spectroscopy measurements.

Section VI: Optical reflection microscopy methods

Cell architecture

Fig. S9: Schematic illustration of the EL-CELL, ECC-Opto-Std test cell assembly used to conduct operando optical reflection microscopy measurements on the HP material during Li ion doping cycles. WE = working electrode, CE = counter electrode.

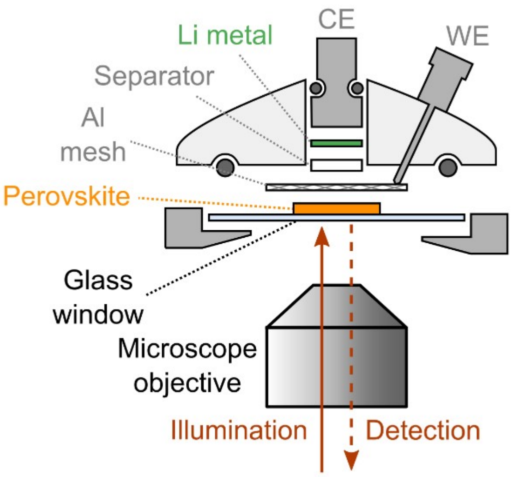

The optical cell (EL-CELL, ECC-Opto-Std test cell) was assembled as per **Fig. S9.** First, the perovskite electrode slurry as described in the main text, was drop cast onto a glass microscope cover slip. The cell stack consisted of the coated cover slip, an aluminium mesh current collector, a glass fibre separator, and a lithium metal chip. Once sealed, the cell was filled with 5M LiTFSI in ethylene carbonate/propylene carbonate (1:1 vol ratio) to act as the electrolyte since the polymer PEO system was not suitable for the EL cell configuration. The window of stability for this electrolyte has been studied in [2] and demonstrates how over the time periods required for this measurement do not show

degradation (dissolution) effects beyond those due to the cycling itself. The aluminium mesh current collector was electrically contacted by a stainless steel pin.

Data acquisition

Images were acquired with a camera exposure time of 2.7 ms, at a frame rate of 10 Hz. Each recorded image was spatially binned (2×2 pixels, giving an effective pixel size of 69.4 nm px^{-1}) and sets of 40 recorded images were temporally binned together to yield an effective frame rate of 0.25 Hz.

Electrochemical control was achieved using a Gamry potentiostat (Interface 1010). Image acquisition and synchronisation of instruments was performed using in-house developed LabVIEW routines.

Differential image analysis

The recorded image stacks were first corrected for stage drift in the *xy*-plane by selecting a stationary sub-diffraction limited spot and fitting its position over time using a two-dimensional Gaussian function. The extracted centre positions in *x* and *y* for each image were subsequently used to correct for stage drift.

Sequential differential images (at each time *t*) were obtained by dividing the pixel values of pairs of frames separated by a time interval (Δt = 40 s) according to:

$$\text{Differential image } (t) = \frac{\text{Image}(t)}{\text{Image}(t-\Delta t)} - 1. \qquad (1)$$

The resulting image contrast displays the fractional intensity change between images, with the contrast centred around 0. This removes constant or slowly varying background contributions and inhomogeneities in the sample illumination, isolating more rapid changes during the interval between pairs of images (40 s). Negative pixel values indicate a reduction in intensity, while positive values indicate an increase, between the two frames.

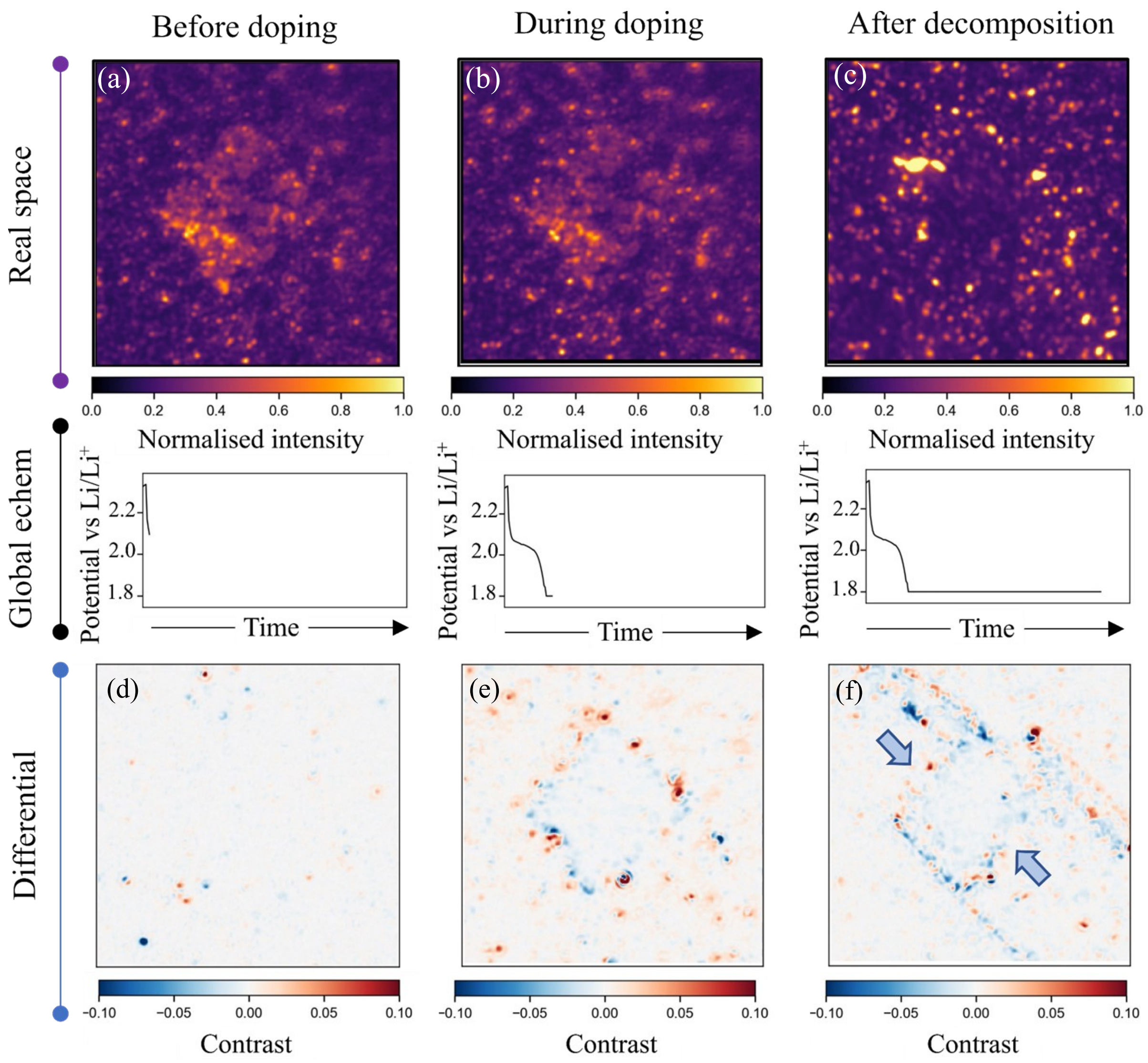

Fig. S10: Optical reflection microscopy images illustrating the structural collapse of the n=4 perovskite form under Li ion insertion. Top row: Bright field scattering images taken using a high intensity 740 nm LED probe. **Bottom row:** Differential images showing a snapshot of the changing reflectivity. **Middle row:** The state of charge during the discharge cycle, corresponding to each image is shown in the panels above and below. NB that this corresponds to the global state of charge and cannot reliably be attributed to the local state of charge of the imaged particle. **(a)** Optical image showing a characteristic square-like platelet of perovskite just before the onset of Li insertion (as the potential drops below 2.2 V). **(b)** After partial lithiation of the electrode via electrochemical doping and **(c)** after the maximal amount of Li ions have been inserted into the electrode – the initial perovskite structure is no longer observed and bright spots of metallic lead appear. The outwards-in loss of perovskite structure is emphasised by considering the differential changes between frames, shown in **(d - f)**. Blue colouration refers to a loss in reflectivity and red to a gain. Therefore, the inward motion of the blue signal refers to the loss of the perovskite, starting from the edges (regions exposed principally to Li ions).

Section VII: Perovskite synthesis materials

Material	Description
**N N-Dimethylformamide anhydrous (DMF)**	99 % purity, anhydrous, Sigma Aldrich
**N-Butylammonium Bromide (BABr)**	98 % purity, Sigma-Aldrich
**Methylammonium bromide (MABr)**	>=99 % purity, anhydrous, Sigma-Aldrich
**Lead bromide ($PbBr_2$)**	98 %, Scientific Laboratory Supplies

Table S2: List of perovskite precursor materials used.

Section VIII: Custom temperature-controlled X-ray Pouch Cell

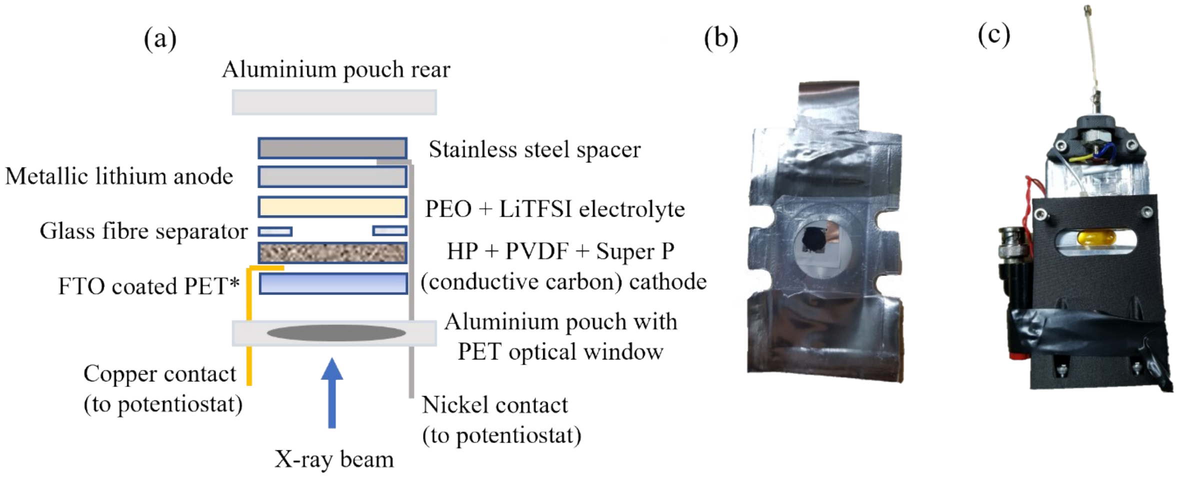

Fig. S11: Bespoke pouch cell for operando XRD measurements. (a) Schematic representation of the battery cell design used for operando XRD measurements. **(b)** Digital photograph of the as assembled pouch cell prior to electrochemical doping and XRD measurements. **(c)** High pressure, high temperature cell casing used to apply externally controlled stack pressure to the cell and uniformly heat the device to the glass transition temperature of the PEO (70 °C).

Fig. S11 Shows a schematic representation of the LIB-inspired material stack used in the X-ray pouch cell used for the operando X-ray experiments of the main text [3]. First, the perovskite electrode slurry (containing the target perovskite phase, super P and PVDF in an 85:10:5 mass ratio) is drop cast onto an indium tin oxide (ITO) coated polyethylene terephthalate (PET) substrate. A glass fibre separator is cut such that it forms a hollow square shape (providing separation between the electrodes via the walls, but allowing full contact with the PEO in the gap) and is placed on top of the electrode. The polymer electrolyte is then cut to size and placed on top of the electrode/separator stack. Finally, the anode (Li metal chip) is attached to a stainless steel disc which is held on top of the rest of the stack. A copper contact is placed on the ITO surface near to the perovskite electrode and a nickel contact is placed on the stainless steel disc. These contacts are allowed to protrude from the pouch cell, once sealed, in order to be connected to the potentiostat. The aluminium pouch cell is then heat sealed and is

held together by the adhesive of the internal surfaces. All of the above steps are carried out under an inert argon atmosphere (< 0.5 ppm O_2, < 0.5 ppm H_2O).

Before being placed into the beamline, the pouch cell is mounted in a custom 3D printed casing which uses a nitrogen “bubble” formed by a Kapton window connected to a gas feed to apply a controlled stack pressure of (~2 bar). The casing also utilizes a copper thermal strip attached to a voltage supply and wrapped around the pouch to heat the pouch to the required temperature. The polymer electrolyte must be heated to above its melting temperature (~70 °C) in order to facilitate significant ionic conductivity [4]. The cell is then placed in the beamline, once connected to the potentiostat for electrochemical control, for the operando X-ray experiments.

Section IX: X-ray single peak analysis

Fig S12: XRD spectra reduction and single peak analysis stages. (a) Reduced 2D spectral data following pyFAI reduction. [5] **(b)** Isolation of a single spectra for a specific spot on the electrode. **(c)** Identification of individual species present in the spectrum. **(d)** Linear background calculation for individual peak and integration *via* gaussian peak fitting to extract peak intensity. Process automated and repeated for every peak and every spot of the electrode.

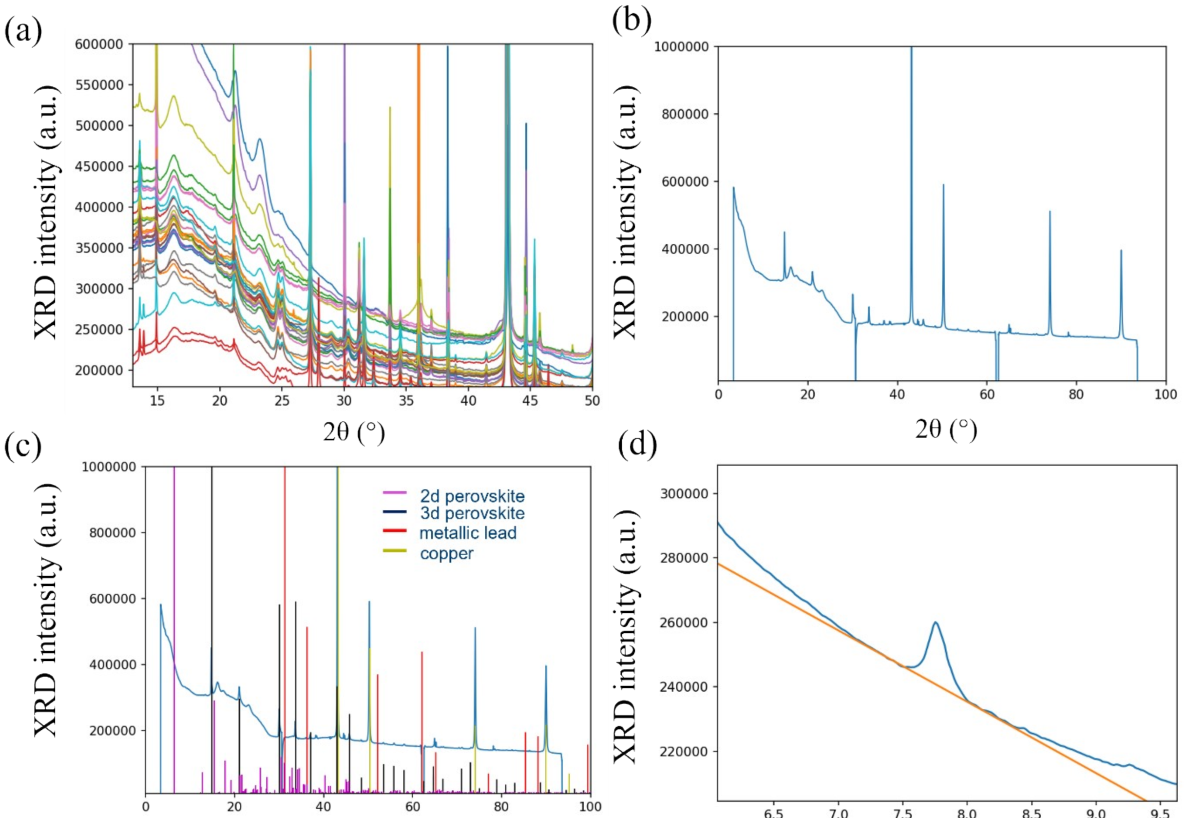

XRD characterisation is undertaken by mounting the custom built XRD pouch cell at SSRL/SLAC beamline 10-2, wavelength $\lambda = 0.9918$ Å (12.5 keV). The potential was controlled using a Biologic SP-150e potentiostat in galvanostatic (constant current) charge discharge mode at a current loading of 60 mA g^{-1}. The diffraction peaks known to belong to the cubic phase of the perovskite are identified and tracked in situ to infer any changes to the perovskite structure as a result of lithium ions being inserted and removed by the external potentiostat. Furthermore, diffraction peaks associated with other known electrochemically inactive species within the cell are observed, such as the copper of the current collector in order to serve as an internal standard from which to calibrate the additional peaks. New peaks, that are not present inherently in uncycled cells are identified and tracked – for example, those belonging to lead and lead bromide. By tracing the position and relative intensity of all peaks, a picture can be established with regards to the structure and species present and how they are dynamically affected by the insertion and removal of lithium. The following processes were automated using home-built scripts based in Python. The first step is the reduction of the 3D raw spectra to a 1D linear spectra this was done using pyFAI following the python module developed by Giannis Ashiotis et al. [5] .

Second, since the X-ray beam was scanned over 30 positions on the sample to avoid damaging the perovskite from over exposure to the high energy photons, the spectra for each location was siloed into a single set. (Stages shown in **Fig. S12 (a - b)**). Individual spectra were then examined and each peak indexed to known species by comparison with the relevant cif powder patterns (**Fig. S12 (c)**).

To extract how the peak intensity varied with the state of Li doping, a background was determined as the linear subtraction between the two points of intersection of the peak. This was then subtracted locally from each peak (example shown in (**Fig. S12 (d)**). This had to be done since the background variation was too great between each spectra to remove the entire background reliably. The remaining y-values are integrated between the intersection points of the peak by fitting a gaussian profile to the peak. These values were then fed back via the time stamp recorded by the XRD detector to the state of charge determined by the potentiostat.

Although the data reported in the main body of the manuscript are representative of a single spot, the analysis was repeated for each spot, in which the perovskite signal was present in the fresh sample, to ensure reproducibility and the validity of the claims.

References

[1] A. Le Bail, H. Duroy and J. Fourquet, "Ab-initio structure determination of $LiSbWO_6$ by X-ray powder diffraction," *Materials Research Bulletin,* vol. 23, no. 3, pp. 447-452, 1988.

[2] A. Mathieson, M. Rahil, Y. Zhang, W. M. Dose, J. T. Lee, F. Deschler, S. Ahmad and M. D. Volder, "Ruddlesden Popper 2D perovskites as Li-ion battery electrodes," *Materials Advances,* vol. 2, p. 3370–3377, 2021.

[3] C. Cao, H.-G. Steinrück, P. P. Paul, A. R. Dunlop, S. E. Trask, A. N. Jansen, R. M. Kasse, V. Thampy, M. Yusuf, J. N. Weker, B. Shyam, R. Subbaraman, K. Davis, C. M. Johnston, C. J. Takacs and M. F. Toney, "Conformal Pressure and Fast-Charging Li-Ion Batteries," *Journal of The Electrochemical Society,* vol. 169, p. 040540, April 2022.

[4] D. T. Hallinan and N. P. Balsara, "Polymer Electrolytes," *Annual Review of Materials Research,* vol. 43, p. 503–525, July 2013.

[5] G. Ashiotis, A. Deschildre, Z. Nawaz, J. P. Wright, D. Karkoulis, F. E. Picca and J. Kieffer, "The fast azimuthal integration Python library: pyFAI," *Journal of Applied Crystallography,* vol. 48, p. 510–519, March 2015.
